# A fragment based approach towards curating, comparing and developing machine learning models applied in photochemistry

**DOI:** 10.1039/d5sc05615b

**Published:** 2025-10-15

**Authors:** Raúl Pérez-Soto, Mihai V. Popescu, Sabari Kumar, Leticia A. Gomes, Changyeob Lee, Elijah Shore, Steven A. Lopez, Robert S. Paton, Seonah Kim

**Affiliations:** a Department of Chemistry, Colorado State University Fort Collins CO 80523 USA s.lopez@northeastern.edu; b Department of Chemistry and Chemical Biology, Northeastern University Boston MA 02115 USA robert.paton@colostate.edu seonah.kim@colostate.edu

## Abstract

The development of graph neural networks for predicting molecular properties has garnered significant attention, as it enables the correlation of quickly computable atomic and bond descriptors with overall molecular properties. With the rising interest in photochemistry and photocatalysis as sustainable alternatives to thermal reactions, curation of virtual databases of computed photophysical properties for training of machine learning models has become popular. Unfortunately, current efforts fail to consider the exciton localization onto different chromophores of the same molecule, leading to potentially large prediction errors. Here we describe a molecular fragmentation strategy that can be used to overcome this limitation, while also providing a way to compare structural diversity between different libraries. Using a newly generated database of 46 432 adiabatic S_0_–T_1_ energy gaps (ALFAST-DB), we compare its diversity with two datasets from the literature and demonstrate that a fragment-based delta learning approach improves model generalizability while achieving accuracies comparable to those of traditional message passing graph neural network architectures (MPGNN).

## Abstract 2

In light of the development of new machine learning models for photochemical property prediction, we show that model development and database construction should focus on chromophore moieties for good extrapolation across chemical space.

## Graphical abstract

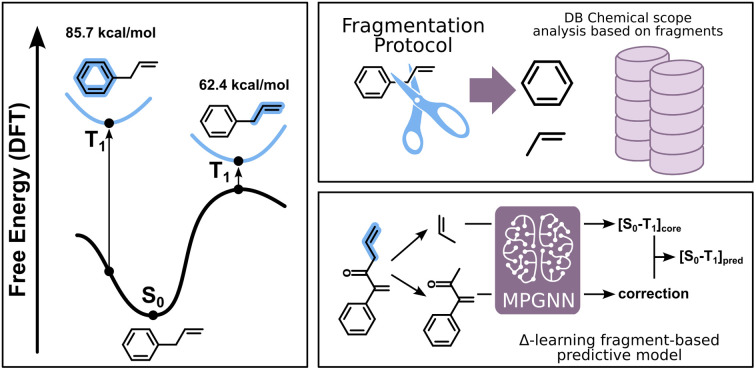

## Introduction

With the re-emergence of photochemistry and photocatalysis as a major driving force of organic chemical synthesis at the start of the 21^st^ century,^1–6^ the development of highly accurate computational methods for the prediction of photophysical properties has been deemed an essential driving force for the acceleration of the discovery of new chemical reactivity and development of new materials.^7–11^ More recently, however, the development of machine learning-based approaches has emerged as a promising alternative to highly computationally expensive quantum mechanical methods for predicting chemical properties, which are then used as an alternative to experimental data.^12–15^ One reason for the rise of machine learning in the last two decades has been an increase in the amount of data available, along with easier access to the most novel model architectures. However, the reliability of any model is highly dependent on the quality of the data used to formulate the model. Thus, the time-consuming step of data curation and validation becomes the rate-determining step in model development.

Due to the difficulties associated with collecting experimental data, models have relied on the construction of large datasets from computational simulations, based on methods such as Density Functional Theory (DFT).^16^ This approach sidesteps several of the limitations associated with data collection, quality assurance, and data interpretation. A plethora of models trained on such computational datasets have been successfully developed, with applications in various fields, including solubility,^17,18^ bond dissociation energy,^19–21^ synthesizability,^22^ and prediction of catalytic efficiency.^23,24^ Thanks to the relative ease of constructing these virtual datasets, many research groups have constructed independent datasets for model development, frequently competing to simulate the same properties. This in turn raises a key question: how can we compare the diversity and quality of competing datasets beyond just the numerical range of the property in question? How can we enhance new datasets by leveraging existing ones to cover a broader range of the chemical space?

One such area of high interest to both the experimental and theoretical communities involves the development and curation of large datasets of photophysical properties. Specifically, within the context of both photoredox and energy transfer catalysis, knowledge of the catalyst and substrate triplet energies is essential for calculating excited-state redox potentials. Also, it facilitates the prediction of reactions amenable to Dexter energy transfer. Within this regard, DFT computation of the adiabatic triplet energy gap (*i.e.*, the thermodynamic driving force between S_0_ and T_1_ state) has become a central method for determining molecular triplet energies, largely due to the experimental challenges involved. To this extent, the adiabatic triplet energy has become a key photophysical property, featured in numerous databases, such as the Verde Materials DB.^25^ Similarly, Glorius and coworkers have recently published the first machine learning model, EnTdecker, for predicting the adiabatic S_0_–T_1_ value, trained on an in-house-generated DFT dataset of 34 848 molecules.^26^ In this work, not only do they demonstrate that Graph Neural Network models outperform other simpler models, but also showcase the practical application of the developed models and build a user-friendly platform for chemists to use their models.

However, as demonstrated in this work, high-throughput DFT calculations of adiabatic S_0_–T_1_ gaps pose challenges when applied to molecules with multiple functional groups. In such cases, exciton localization can vary semi-randomly across different regions of the molecule, leading to significant energy differences and introducing substantial error in model construction. In this work, we present a new fragmentation algorithm designed to address this issue. This algorithm enables the training of a *Δ*-learning model on a new dataset of 46 415 molecules (ALFAST-DB), following a similar protocol to EnTdecker. Additionally, the algorithm offers a novel approach to comparing the chemical compositions of different databases and evaluating the transferability of ML models trained on them.

### Exciton localization problem in multichromophore systems

As highlighted earlier, DFT optimizations on excited states are particularly challenging for molecules with multiple chromophores due to the issue of exciton localization. Taking allylbenzene as an example, the standard approach begins by using the optimized geometry of the molecule in its ground state (S_0_) as the starting point for optimizing the corresponding triplet state (T_1_). The geometry optimization on the T_1_ surface proceeds from this initial geometry, and the algorithm directs the structure toward the nearest local minimum based on the gradient of the surface curvature.

**Fig. 1:**
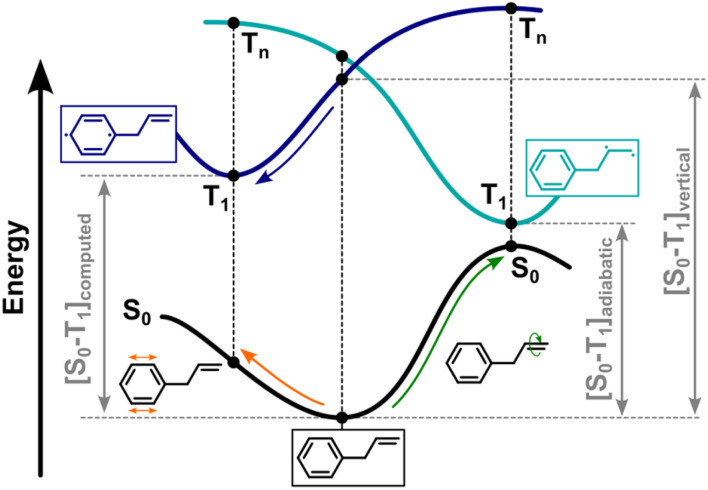
Diagram describing the computational structural optimization on the T_1_ surface starting from a ground state geometry.

In the case of allylbenzene, this initial optimization leads to a structure where the triplet diradical localizes on the phenyl group, as illustrated in Fig. 1. However, this localization isn't always unique. If the molecule is initially excited to a higher triplet state (T_*n*_) instead, the system may follow a different potential energy surface. This new surface can intersect the initial T_1_ surface and, in some cases, drop below it in energy, effectively becoming the new T_1_ state. Such a scenario results in the localization of the spin density on a different region of the molecule.

For allylbenzene, the system can alternatively find a T_1_ minimum where the spin density localizes on the alkene group. While the optimization process preferentially leads to the excitation of the aryl ring, this alkene-localized minimum is also a valid solution. It is energetically more favorable, resulting in a lower adiabatic triplet energy. In this case, when computed at the M06-2X/def2-TZVP level using Gaussian 16,^27^ the adiabatic triplet energy (62.4 kcal mol^−1^) is lower by an impressive 23.3 kcal mol^−1^ compared to the phenyl-localized minimum (85.7 kcal mol^−1^).

This difference in localization presents a significant challenge for automated excited-state optimization in database construction. In systems with multiple chromophores, it becomes nearly impossible to predict *a priori* where the triplet state will localize. As a result, this uncertainty complicates the construction of a coherent dataset, often leaving the data effectively “unlabeled” in terms of which chromophore is involved in the triplet state localization. This type of unstructured data introduces confusion into machine learning models, as the models may struggle to learn from or differentiate between cases involving different chromophores. Furthermore, it raises an important question regarding the goal of adiabatic triplet energy predictions: is the objective to predict the global minimum energy state, or should the models aim to predict all possible local minima? Defining this objective becomes crucial in ensuring the predictive power and applicability of the machine learning models.

One potential solution, as implemented by the EnTdecker authors, is to provide a prediction of spin density in conjunction with the adiabatic triplet energy value. This strategy adds valuable information to the user, offering insight into where the triplet localization occurs within the molecule. However, while this represents an important step forward, it does not fully resolve the underlying issue of training models on unlabeled data. Since the spin density localization is not systematically assigned to a specific chromophore or fragment, the model is still learning from data that may not be explicitly categorized.

Moreover, since these spin density models were trained independently of the energy predictions, there is the potential for inconsistencies. In some cases, the energy and spin density predictions may not align, potentially leading to misleading conclusions for the user.

## Results and discussion

### Molecular fragmentation algorithm

**Fig. 2:**
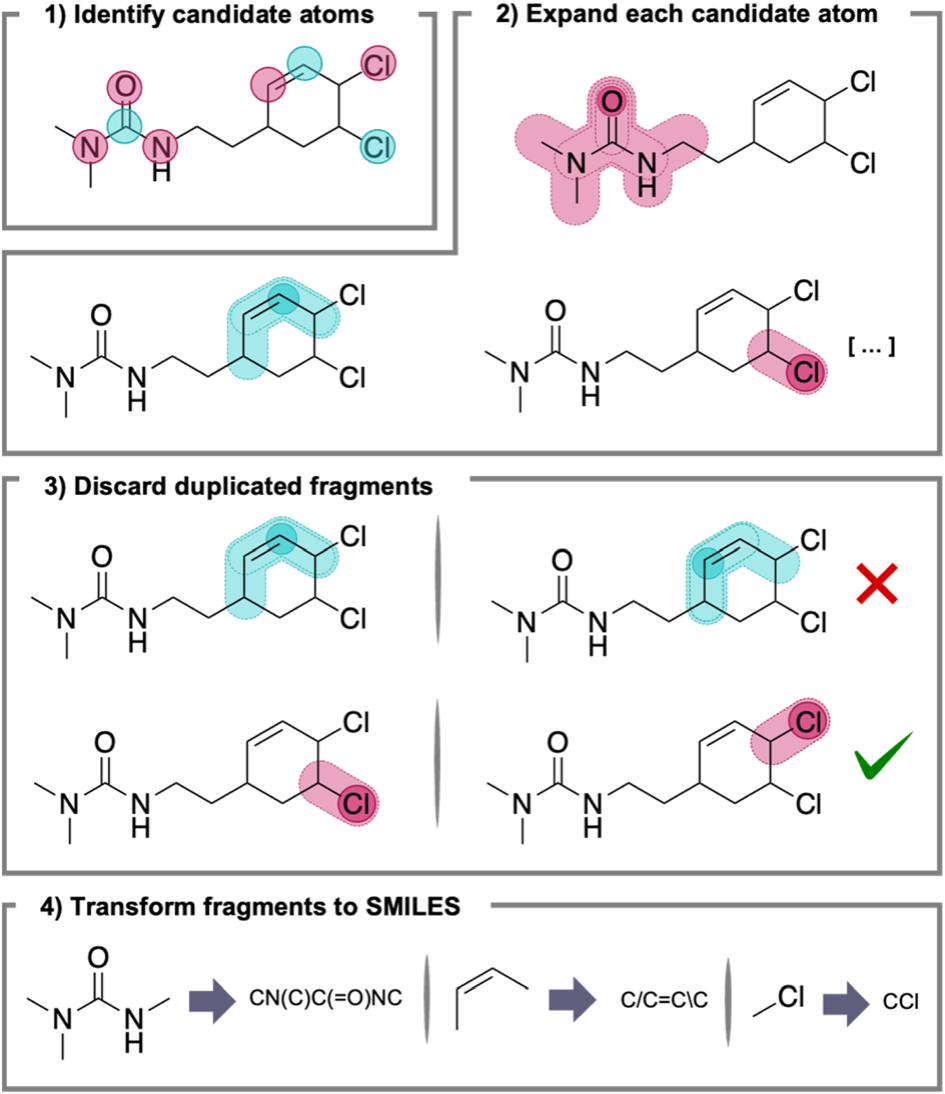
Scheme and example of the application of the fragmentation algorithm used in the present work.

To address the challenges associated with exciton localization and unlabeled data in multi-chromophore systems, a first step is to develop a workflow for identifying potentially relevant sites where the exciton can be localized. Here we developed a fragmentation algorithm that generates chemically meaningful fragments from complex molecules, akin to the one described by Ertl in 2017.^28^ This approach was inspired by the concept of functional groups in organic chemistry, which serve as the building blocks of molecules and represent key sites of chemical reactivity. Functional groups, often composed of π-bonds and heteroatoms linked by resonance, are rich in π-electrons and lone pairs. These electron-rich regions are frequently the most likely sites for excitations due to their lower energy gaps between π–π* and n–π* orbital levels.^29^ By leveraging this understanding of functional groups, this algorithm systematically breaks down molecules into these reactive components based on extended conjugation paths, leading to more structured data that can be effectively used for machine learning. The steps of the algorithm are illustrated below (Fig. 2), and include the following key processes:

(1) Identify all relevant heteroatoms based on a predefined list, as well as all carbon atoms containing a double, triple, or aromatic bond. Specifically for this work, we considered the following candidate heteroatoms: N, O, S, P, Se, F, Cl, and Br.

(2) For each candidate atom, a list is constructed that contains these atoms. Then, the atoms directly bonded to each candidate atom are recursively added. If the connecting atoms are also candidate atoms, the process continues to add their connected neighbors until no candidate atoms are found. This recursive process is stopped when either there are no more atoms or the last atom added was a non-candidate atom.

(3) Once no candidate atoms remain in reach of the molecule, duplicated fragments are removed.

(4) Finally, for database storage analysis and curation, each fragment is transformed into its SMILES representation. During this step, the end-atoms of the fragments are implicitly capped with H to match their valence. As all valid SMILES are valid SMARTS, H-capping allows the generation of smaller, valid, typically closed-shell molecules containing the key functionality of the fragment.

Following the example highlighted above (Fig. 2), the outcome of the fragmentation scheme as applied to 3-(2-(4,5-dichlorocyclohex-2-en-1-yl)ethyl)-1,1-dimethylurea will lead to the identification of four distinct functional groups, namely: a trisubstituted urea, a disubstituted (*Z*)-alkene, and two alkyl chlorides.

### Databases overview and curation

**Fig. 3:**
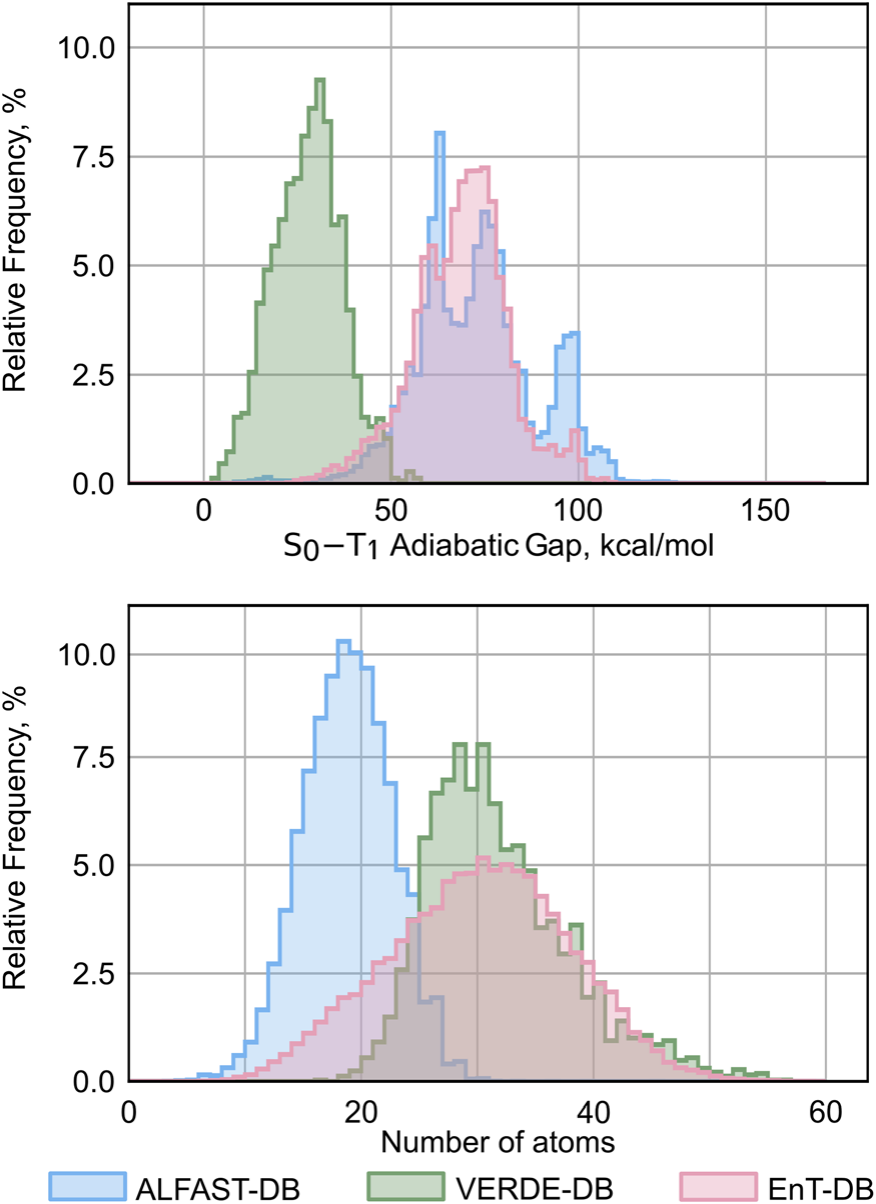
Total number of atoms and S_0_–T_1_ adiabatic gap distributions of the 3 presented datasets.

The present work utilizes three separate datasets comprising adiabatic S_0_–T_1_ energy gaps. One such dataset is the EnT-DB database, developed by Glorius and coworkers, which was used to develop the EnTdecker predictive model. The EnT-DB dataset consists of DFT-computed adiabatic S_0_–T_1_ energy gaps for a total of 34 848 molecular species. Energies are computed at the ωB97x-D3/def2-TZVPP:PCM(CH_3_CN)//B3LYP-D3/def2-SVP:PCM(CH_3_CN)^30–35^ level of theory, using the ORCA software package.^36^ Molecules were selected to maximize chemical space coverage of medicinally relevant structures, and were selected from the ZINC dataset^37^ clade of commercially available species with molecular weights under 325 Da. The size distribution of molecules in the dataset is roughly symmetric, with a median of 30 atoms. Calculated adiabatic S_0_–T_1_ gaps span a broad range, with a median of 69.4 kcal mol^−1^ (Fig. 3, EnT-DB).

The second database used in this work is the Verde Materials Database previously developed by Lopez and coworkers (Verde-DB). The version of the dataset used here is more comprehensive than the one previously published; this version of the Verde-DB dataset contains 3286 molecules, compared to the 1500 molecules in the original work. The S_0_–T_1_ adiabatic gaps in this database are also obtained using DFT, but computed at the M06/6-31+G(d,p):IEFPCM(CH_3_CN)^38–42^ level of theory. In contrast to EnT-DB, which focuses solely on adiabatic S_0_–T_1_ gaps as a target property and prioritizes a broad chemical scope, Verde-DB is more focused on providing broader coverage of properties related to photoredox catalysis, such as excited-state reduction and oxidation potentials, or S_0_–S_1_ energy gaps, at the cost of a narrower chemical scope. This chemical scope is more focused on molecules used in commercial applications, with additions of computationally generated derivatives. The molecular size distribution (Fig. 3) has the same median as the EnT-DB dataset (30 atoms), while showing a pronounced tail towards larger molecules. Due to the specialized chemical scope targeted in the Verde-DB dataset, it exhibits a strong bias towards molecules with a small S_0_–T_1_ adiabatic gap, with a median value of 28.2 kcal mol^−1^.

Finally, we develop our database targeting a broad chemical scope, following a protocol where the T_1_ geometry is optimized using the optimized ground state structures as a starting point. Previously, we compiled a large database of DFT-computed structures for predicting bond dissociation enthalpies, which led to the creation of the ALFABET model. The ALFABET dataset was designed to cover a broad range of organic chemical space and comprises molecules with atoms of C, H, N, O, S, Cl, F, P, Br, and I. Building on this previous work, we compute the S_0_–T_1_ adiabatic gaps for molecules of interest in this database. This initial set comprises 57 736 molecules after removing radicals and retaining only those molecules with double, triple, or aromatic bonds. For the level of theory used in computation, we employ the M06-2X/def2-TZVP level of theory, in contrast to the one used to construct the EnT-DB dataset. Although the M06-2X functional had been identified as optimal for adiabatic triplet energy calculations by the Glorius group, challenges with convergence and computational costs led to the ωB97x-D3 functional being used instead. While recent work suggests that benchmarking of adiabatic triplet energies against experimental measurements may not be representative of physical reality and can lead to very large errors on systems that undergo high structural reorganizations, benchmarking against the adiabatic values computed at the “gold standard” DLPNO-CCSD(T) method with the cc-PVTZ basis set^43–48^ also showed that the M06-2X provides the best accuracy. Furthermore, the S_0_ ground states in the original ALFABET dataset were computed at the M06-2X/def2-TZVP level, allowing us to avoid the significant computational cost mentioned by Glorius and coworkers; we thus computed the T_1_ minima at the same level of theory. Out of the 57 736 starting molecules, 53 137 achieved successful convergence, which were further curated down to 46 415 molecules after further quality assurance checks (see SI for further details). As shown in Fig. 3, this database is more focused on smaller molecules than the EnT-DB or Verde-DB datasets, with a median number of atoms of 18. However, we maintain a similar distribution of S_0_–T_1_ adiabatic gaps to EnT-DB, with a median of 72.6 kcal mol^−1^. We refer to this new dataset as the ALFAST-DB.

### Database analysis and comparison

Using the framework of the proposed fragmentation scheme, we analyze the fragment compositions of the three databases discussed above. First, fragments that did not contain a double, triple, or aromatic bond (using SMARTS pattern matching) were discarded. These fragments were then used to obtain the possible exciton localizations within each molecule across the databases (*vide infra*). This fragment-based breakdown allows for a more quantitative evaluation of the relevant chemical diversity and overlap between the three datasets.

First, we evaluate the fragment overlap between databases (Table 1). We observe that while the ALFAST-DB has a larger number of molecules (46 415 *vs.* 34 848), it has a slightly smaller diversity of unique fragments than the EnT database (15 410 *vs.* 18 909). Between these two databases, there are 2146 shared fragments, while neither database contains shared fragments with the Verde-DB. Next, we analyze the number of fragments formed for each molecule and quantify the percentage of shared fragments within this classification scheme (Table 2). Interestingly, a large number of molecules in each dataset contain a fragment from the small shared subset of 2146 fragments, at 62.6% and 51.6% for ALFAST-DB and EnT-DB, respectively, indicating a significant overlap in chemical space. This overlap is thus more significant than one would expect if only the identity of the complete shared molecules (of which there are 1530) is considered. Almost all unique fragments correspond to single-fragment molecules, and the EnT-DB has a higher composition of multi-fragment molecules (32.4% *vs.* 15.5%). Notably, the Verde-DB is constituted solely of mono-fragment molecules (thus not shown for brevity). We posit that this may be a consequence of the more specialized scope considered when constructing the Verde-DB.

**Table 1:** Number of shared fragments between databases. The diagonal values correspond to the total number of fragments of each database

Databases	ALFAST	EnT	Verde
ALFAST	15 410	2146	0
EnT	2146	18 909	0
Verde	0	0	3286

**Table 2:** Molecule breakdown of the ALFAST-DB and EnT-DB. Molecules are classified as either single-fragment or multiple-fragment, and based on whether at least one of the fragments is within the subset of fragments shared across the two databases (shared) or all fragments of the molecule are unique. Numbers in parentheses are the absolute number of molecules

	ALFAST-DB		EnT-DB	
	Single	Multiple	Single	Multiple
Unique	37.2% (17 289)	<0.1% (35)	47.8% (16 649)	0.6% (212)
Shared (2146)	47.2% (21 941)	15.4% (7150)	19.8% (6911)	31.8% (11 076)
Total	84.5% (39 230)	15.5% (7185)	67.6% (23 560)	32.4% (11 288)

It is important to note that these statistics do not provide a basis for claiming the superiority of one dataset over the other. On the one hand, a larger number of unique fragments can be taken naively to represent increased chemical diversity of the constituent chromophores. On the other hand, a larger ratio of molecules to unique fragments provides a greater diversity of substituent effects on the chromophores, thus potentially improving the generalization characteristics of derived models.

**Fig. 4:**
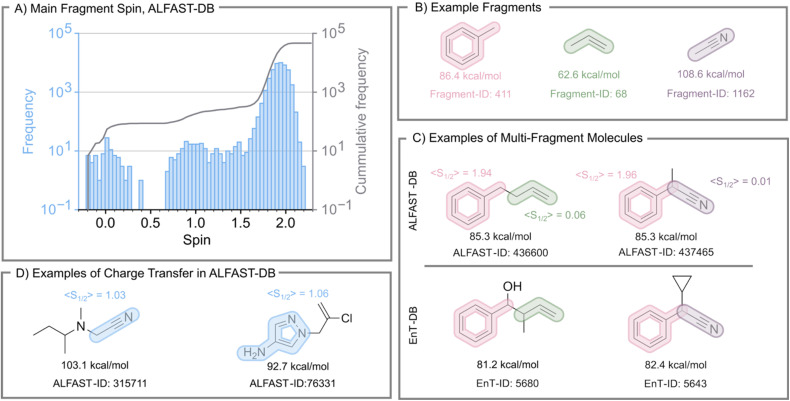
(A) Absolute (blue histogram) and cumulative frequency (grey line) of the distribution of the main fragment's spin within the ALFAST-DB. (B–D) Example DFT-computed S_0_–T_1_ adiabatic gaps for fragments and for multi-fragment molecules within the ALFAST-DB and the EnT-DB. The spin of each fragment was computed from the atomic Mulliken Spin Densities. The example fragments (B) were computed at the same theory level as ALFAST-DB. We note that the EnT-Database does not explicitly include an ID number for each molecule; ID numbers were assigned in this work for organization.

Finally, to assess the effect of exciton localization mentioned previously, we utilize the Mulliken spin densities at the optimized T_1_ states included in the ALFAST-DB to compute the total spin of each fragment obtained. The fragment with the highest spin was then selected as the “main fragment” of the molecule, effectively labeling each molecule with a main fragment, and a total spin of the main fragment. The distribution of these main fragment spins across the ALFAST-DB is shown in Fig. 4A. While the majority of the database has a localized triplet, where the spin of the main fragment is over 1.7, three notable outlier regions require further discussion: molecules that have a main fragment with a spin over 2, molecules where the main fragment spin is between 0.7 and 1.3, and molecules whose main spin is below 0.65. We note that the choice of 1.7 as a threshold for triplet spin localization is an arbitrary value chosen for analysis and discussion of the generated database and should not be construed as a metric to define triplet spin localization. We leave rigorous benchmarking of this metric for future study; all computed values for fragment spins are available in the database files supplied in the Zenodo repository linked in the SI (DOI: https://doi.org/10.5281/zenodo.16563830).

We posit that the first region emerges as an artifact of the truncation during the total spin density calculation of the fragment. The second and third regions, although having more consequential root causes, comprise less than 1% of the molecules in ALFAST-DB (approximately 500 molecules). Thus, their impact on the overall database is considered minor. The second region (between 0.7 and 1.3) corresponds to molecules where a charge transfer state was likely obtained. The third region, however, emerges as molecules whose main fragment was incorrectly assigned. This appears in cases where the obtained minima in the T_1_ surface were not localized in a fragment containing a double, triple, or aromatic bond.

To illustrate this electron delocalization problem further, we analyzed representative examples from each of the outlier regions described above (Fig. 4B and C). Examples were chosen such that the localization of the exciton can be confirmed based on the spin of the fragment. In addition, some example molecules where a charge transfer state was obtained are highlighted (Fig. 4D). As shown in Fig. 4B, the adiabatic S_0_–T_1_ gap of toluene is lower than that of acetonitrile. As such, a molecule containing both moieties separated by an alkyl carbon chain should be able to access the triplet state of both moieties. In the selected examples (ALFAST-DB and Ent-DB, right column, Fig. 4C), the computed value is more similar to the adiabatic gap of toluene. In the ALFAST-DB example, we can confirm that the exciton location is the phenyl ring. However, in this example, the overall high-throughput DFT computational protocol can be considered successful in approximating the adiabatic S_0_–T_1_ gap, which is understood as the lowest free energy difference between the S_0_ free energy surface and the T_1_ free energy surface.

On the other hand, the examples highlighted with phenyl and alkene moieties (ALFAST-DB and EnT-DB, left column, Fig. 4C) show the opposite result: out of the two exciton locations the minima with the phenyl-localized triplet was favored, which is higher in energy than that localized in the alkene (based on the difference between toluene and propene fragments), thus illustrating an unsuccessful application of the high-throughput DFT computational protocol. When the structure obtained from the S_0_ optimization is used as a starting structure for the T_1_ optimization, the resultant T_1_ minima localize the triplet in the phenyl moiety. Instead, the starting structure must be manually distorted to an alkene-like geometry (H–C–C–H dihedral angle of 90°) to localize the triplet on the alkene moiety and obtain a lower energy T_1_ minimum. Further work is necessary to elucidate which factors may bias the geometry optimization towards either of the T_1_ minima, as well as how these factors affect the numerical energy value, in order to develop a high-throughput DFT protocol that finds the “true” lowest energy triplet in an automated manner without requiring manual geometry distortion.

The final charge transfer examples (Fig. 4D) show two different cases. In the rightmost example, there are two clear, disconnected π-systems where the spin is localized, whereas the other example shows a case where not all the spin is localized in a region of the molecule containing a double, triple, or aromatic bond.

### Architecture selection and model extrapolation

Prior work by numerous groups has shown the effectiveness of Graph Neural Network models in predicting molecular properties. Here, we tested various message-passing neural network architectures based on the previously successful ALFABET model (further details are provided in the SI). Model evaluation was performed using a 64 : 16 : 20 train : validation : test split of the ALFAST-DB with randomized splitting. A 5-fold cross-validation strategy was used within the train-validation subsets. The validation loss function (mean absolute error, MAE) was tracked during training and used to select the best model for each validation fold. The final architecture and training strategy presented a cross-validation MAE (aggregated across folds) and test-set MAE (averaged across folds) of 1.94 and 2.00 kcal mol^−1^, respectively. For the final predictions and the presented parity plots, the model was trained using the combined training and validation sets, resulting in a test-set MAE of 1.94. When trained on the EnT-DB using a similar splitting strategy, the cross-validation MAE and the averaged test-set MAE were 2.04 and 2.06 kcal mol^−1^, respectively, with a final model test-set MAE of 2.37 kcal mol^−1^. Compared to the random splitting results of the EnTdecker GNN models, with MAEs of 1.97 and 1.93 kcal mol^−1^ for the Chemprop-D-MPNN and AttentiveFP-GNN models, respectively, it can be observed that our selected architecture achieves similar accuracies, regardless of the database on which it was trained.

**Fig. 5:**
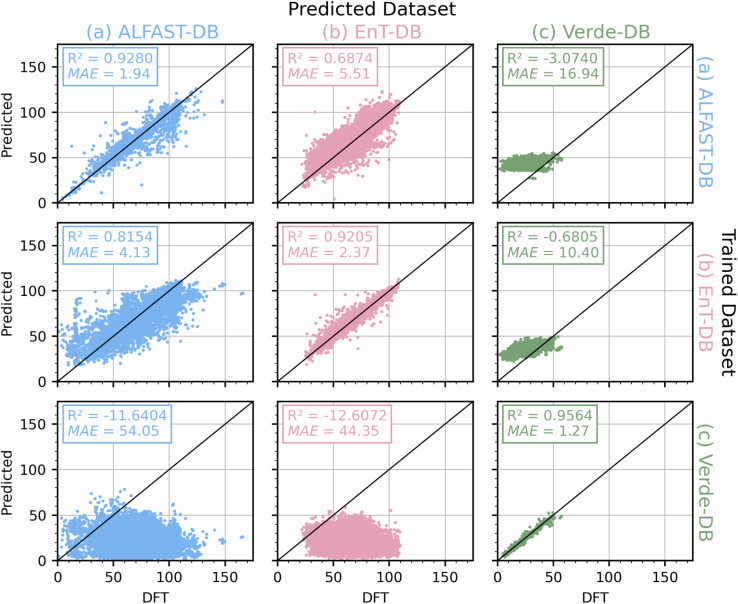
Model extrapolation across datasets. Parity plots of adiabatic S_0_–T_1_ predictions in kcal mol^−1^ of each dataset (column) of the same model architecture trained each dataset (row). For example, parity plot “ab” corresponds to the architecture trained with the combined training and validation set of ALFAST-DB (80% of the database) and shows the prediction of EnT-DB. The predictions on the diagonal plots only shows the molecules in the test set (remaining 20% of the database) of each respective database. The metrics, *R*^2^ and MAE values, correspond to the data presented in each plot. MAE values are in kcal mol^−1^.

After the architecture selection, the performance of the models on the other unseen datasets was evaluated to assess model extrapolation capabilities. As each database is generated using a different DFT level of theory, a degradation in model performance is to be expected. This increase in model error stems from two separate causes: the uncertainty of the ML model in making predictions on unseen data, and the error incurred when comparing S_0_–T_1_ adiabatic values computed using different DFT theory levels. To attempt to separate these two sources of error, each database's final model (trained with the respective combined training and validation sets) was then used to predict the values of each dataset (Fig. 5). The prediction of each final model on its respective test set (Fig. 5aa, bb and cc) provides an estimate of the model's uncertainty. Conversely, the predictions on the complete unseen datasets (Fig. 5ab, ac, ba, ca, bc and cb) provide insight into how the model extrapolates.

From this analysis, two clear trends are observed. First, when the architecture is trained on either ALFAST-DB or EnT-DB (*i.e.*, the datasets with more general chemical space coverage) and used to predict the other (Fig. 5ab and ba), a clear increase in the MAE is observed, while some degree of correlation (marked by *R*^2^) is retained, on the other hand, when the training and prediction are performed using the more specialized Verde-DB and the other two remaining databases (Fig. 5ac, bc, ca and cb) respectively, the models fail drastically at extrapolation. This behavior reproduces known trends regarding the importance of training set chemical space diversity on model performance.

To estimate the magnitude of the error incurred when comparing values computed with different DFT functionals, we analyze shared molecules between ALFAST-DB (in gas phase) and EnT-DB (in acetonitrile). For this task, the canonical SMILES representations of molecules from both databases were obtained and used to search for common molecules, of which 1530 were identified. An MAE and *R*^2^ of 3.36 kcal mol^−1^ and 0.88, respectively, were obtained when comparing the S_0_–T_1_ adiabatic gap of these 1530 molecules (see Fig. S7 in the SI); a linear fit was also performed, yielding an MAE and *R*^2^ of 2.63 kcal mol^−1^ and 0.92, respectively (see Fig. S7 in the SI). It should be noted that this MAE represents errors derived from directly comparing the results of different DFT methodologies, but also molecules where each method computed a different exciton location, molecules whose exciton is located on the same fragment but geometry optimizations led to different local minima, or molecules that would be more drastically stabilized in the triplet state by the acetonitrile solvent.

If then, the uncertainties in both model predictions and the underlying DFT methodology are combined, the model trained on ALFAST-DB achieves a net uncertainty of 5.30 kcal mol^−1^, which is comparable to the MAE of 5.51 kcal mol^−1^ obtained when using the same model to make predictions on the EnT-DB (Fig. 5ab). The same can be observed when the model trained on the EnT-DB extrapolates to the ALFAST-DB. This observation stays the same even when the cross-validated MAEs of the models are considered (1.94 and 2.04 kcal mol^−1^ for ALFAST-DB and EnT-DB, respectively). We note that the combination of both errors provides an approximation to the actual contribution to the error of the cross-dataset prediction. When we corrected the S_0_–T_1_ predicted using the MPGNN model with the linear fit derived from the shared molecules, a decrease in the MAE (Fig. S8 in the SI), although limited, can be observed.

In contrast, we observe that such “symmetric” behavior is not evident when considering the Verde-DB. As seen from the analysis above, the Verde-DB shares no fragments nor molecules with either of the other two databases, is constituted only of mono-fragment molecules, and is an order of magnitude smaller than the other two databases due to its more specialized nature.

**Fig. 6:**
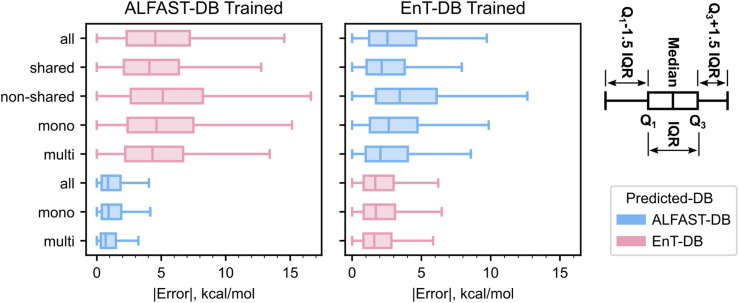
Boxplots with total (all) and marginal absolute error distributions of molecules based on whether they have a shared fragment between databases (shared *vs.* non-shared) or whether they are mono- or multi-fragmented molecules (mono *vs.* multi). *Q*_1_ and *Q*_3_ are the first and third quartile respectively and IQR stands for interquartile range. The error distributions of the dataset used for training are the distributions of the database's test set, while the distributions for the extrapolated database include the full extrapolated database.

Both the lack of shared fragments and the number of mono-fragment molecules may be responsible for the poor extrapolation to and from Verde-DB. To provide further insight into this, a detailed breakdown of errors in the ALFAST-DB-trained and EnT-DB-trained models (Fig. 6) based on fragment presence was obtained.

First, it is reaffirmed that the absolute errors are significantly smaller and the distribution is narrower (Fig. 6) when predicting the dataset on which the model was trained, as compared to the database to which it is being extrapolated. However, a clear difference is evident between the shared and non-shared fragment error distributions of the extrapolated dataset (Fig. 6), where molecules without a shared fragment exhibit a larger error than those with a shared fragment. This involves a shift of the median of approximately 1–2 kcal mol^−1^ and a broadening of the error distribution. On the other hand, such consistent effects are not observed between mono- and multi-fragment distributions; interestingly, both models exhibit slightly better performance for multi-fragment molecules than for mono-fragment molecules, regardless of the predicted dataset.

One interpretation of this trend is that the model better fits multi-fragment molecules, as they are more information-dense. If indeed this is the case, the model should also exhibit lower errors for molecules that suffer from the exciton localization problem discussed earlier. It is to be noted that this interpretation assumes that the differences between the mono-fragmented and the multi-fragmented error distributions are not an artifact of the subset size (see molecule breakdown in Table 2), nor due to the lower chemical diversity of the main fragments in the multi-fragment molecules. Nonetheless, the consistent difference when extrapolating trained models to molecules with shared fragments *vs.* molecules with non-shared fragments, and the poor extrapolation to and from Verde-DB point towards the shared-fragment overlap as the predominant cause of the large prediction errors.

It is thus clear that one method to improve the generalizability of a model trained on a dataset of properties dependent on the exciton location is to increase the chemical scope of the fragments, instead of increasing the frequency of the fragments already present in the dataset.

### Fragment-based models

As fragment overlap between different datasets appears to directly correlate with the model's extrapolation ability, at a first-level approximation, it may be assumed that expanding the datasets with more fragments or even using the fragments as a surrogate prediction for the entire molecule may provide an interesting approach towards improving model performance.

To explore this angle, we created a dataset dubbed Fragments-DB which consists of DFT-computed adiabatic S_0_–T_1_ gaps for each fragment present in ALFAST-DB or EnT-DB. We applied the fragmentation algorithm and included entries that appear at least twice in either of the two datasets, resulting in a total of 6058 unique fragments. Following the same protocol for constructing ALFAST-DB, we obtained a total of 5135 S_0_–T_1_ adiabatic gaps for structures in Fragments-DB.

**Fig. 7:**
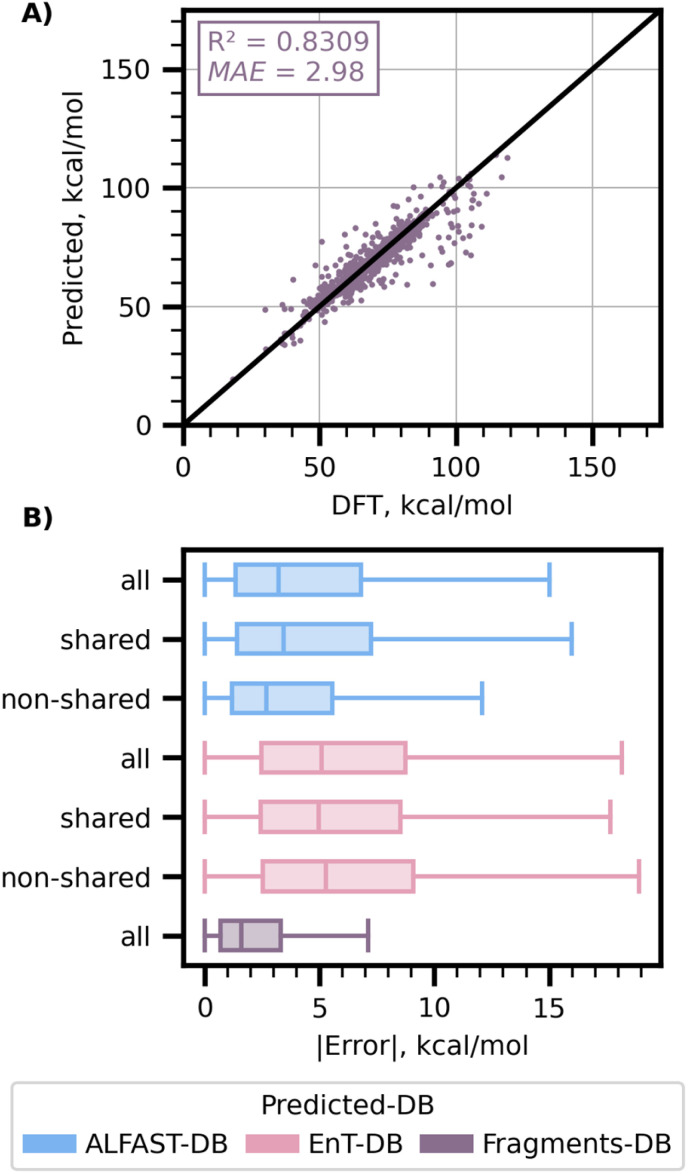
(A) Predicted *vs.* computed S_0_–T_1_ adiabatic gaps of the model trained on the Fragments-DB. (B) Boxplots with total (all) and marginal absolute error distributions of molecules based on whether they have a shared fragment in Fragments-DB (shared *vs.* non-shared).

Using this new Fragments-DB, we retrained the GNN model following the same approach as employed prior (Fig. 5) to allow for direct comparison with the larger ALFAST dataset. Following this approach, the resulting GNN model performed slightly worse, yielding a test-set MAE of 2.98 kcal mol^−1^ and a coefficient of determination of 0.83 (Fig. 7A).

The decrease in predictive accuracy compared to the ALFAST-DB trained GNN (Fig. 5aa, 1.94 kcal mol^−1^) is attributable to the composition of Fragments-DB. It is to note that due to the construction of Fragments-DB, only one instance of each fragment is present in the training set. Thus, a random splitting strategy does not have the same effect on model training with Fragments-DB as with ALFAST-DB. The accuracy presented here should hence be evaluated against the 3.12 kcal mol^−1^ MAE of the out-of-sample splitting results presented by Glorius.^26^

**Fig. 8:**
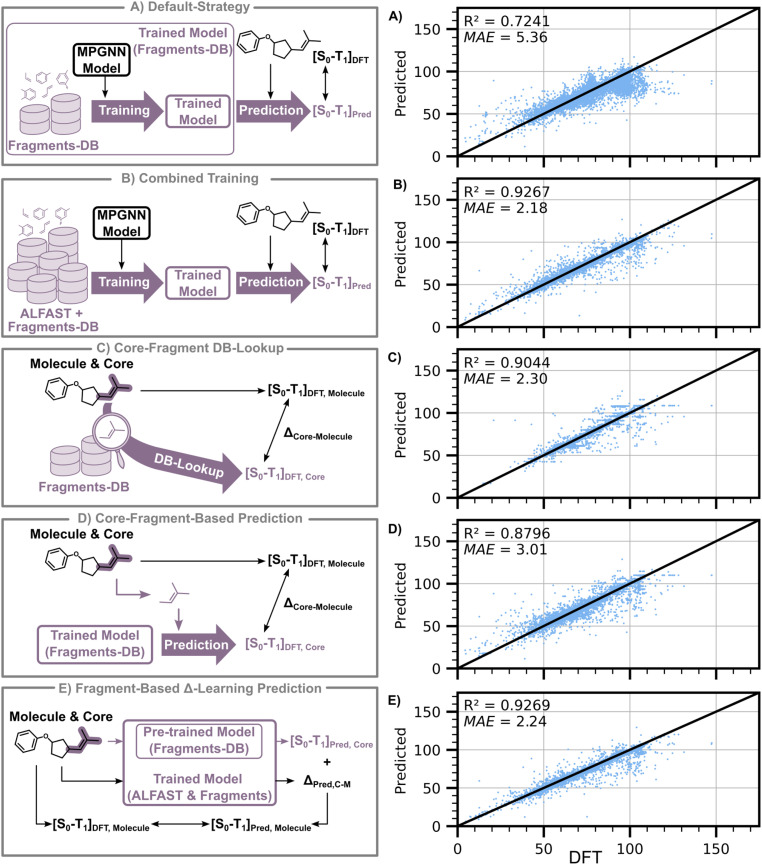
General illustration of the different “prediction” schemes discussed ((A)–(E) left) and their corresponding parity plots on the test set of ALFAST-DB ((A)–(E) right). All presented numbers are in kcal mol^−1^ except the *R*^2^ coefficients which are dimensionless. In this figure “Core” is used to refer to the fragment where the exciton is localized. Reported MAE and *R*^2^ are the metrics of the predictions of the ALFAST-DB test set.

Intriguingly however, when the Fragments-DB-trained model was applied to predict the test set of ALFAST-DB, the *R*^2^ and MAE were found to be 0.72 and 5.36 kcal mol^−1^, worse than the Fragments' test set MAE (2.98) or its cross-validation values (MAE of 3.07 and 3.26 for the aggregated validation and test set respectively, see SI). We then attempted to investigate the cause of this discrepancy by analyzing the errors in the context of fragment overlap (Fig. 7B). Notably, the errors remain consistent between shared and non-shared fragments, for both ALFAST-DB and EnT-DB. There are several possible explanations for this behavior: (a) the degradation in performance may be due to the excitation localization problem discussed previously, as the fragment based model is trained on individual chromophores only, and thus multi-chromophore molecules are out-of-distribution; (b) the model may be failing to learn contributions from the whole molecule (*vide infra*, Fig. 8D); (c) the model fails at representing large molecules; (d) it is known that certain functional groups more readily optimize to triplets than others (*e.g.* aryl groups); thus, it may be that the whole-molecule ALFAST model simply learns the identity of these groups more readily than the fragment model. We leave further investigation of these possibilities to future work.

To address the exciton localization problem while exploring the origin of deviations between these models, we employed a series of new training strategies that systematically consider the contributions of the fragments. We initially investigated the simple strategy of dataset augmentation, where ALFAST-DB was naïvely augmented using Fragments-DB (Fig. 8B), keeping only the entry from the Fragments-DB when a molecule was present in both databases. When trained on the combined training and validation sets of each database, a test-set MAE (evaluated only on the test-set of ALFAST-DB) of 2.18 kcal mol^−1^ and an *R*^2^ of 0.93 was obtained (cross validation results and performance on the combined test-set are provided in the SI). When compared with the model trained on whole molecules from the ALFAST-DB alone (MAE of 1.94, Fig. 5aa), a slight decrease in performance is observed. The resulting 11% degradation is attributable to the differing chemical spaces represented by ALFAST-DB and Fragments-DB: the Fragments-DB is composed of smaller constituent chemical species and thus presents a different input space distribution than the ALFAST-DB. Nevertheless, this new model forms a more robust baseline for performance comparison.

We next evaluated the incurred error when the adiabatic S_0_–T_1_ gap of the molecule is approximated as the adiabatic S_0_–T_1_ gap of the main fragment. As such, we developed a “core-fragment DB lookup scheme”, where we approximate the value of the S_0_–T_1_ gap for the whole molecule to be same as the DFT-computed value of the minimal size representation of the fragment where the excited triplet is localized (Fig. 8C), providing an estimate of the error only due to the fragmentation approximation. Subsequently, the “core-fragment prediction scheme” employs a similar methodology; however, a Fragment-DB-trained GNN model is used for prediction instead of DFT (Fig. 8D). Interestingly, for the GNN-based approach, the ALFAST-DB test set MAE is reduced from 5.36 to 3.01 kcal mol^−1^ by merely switching the target of the prediction from the whole molecule (Fig. 8A) to the main fragment of the molecule (Fig. 8D). The MAE further decreases to 2.30 kcal mol^−1^ when the DFT-calculated fragment adiabatic S_0_–T_1_ gaps are used (Fig. 8C). This observation is suggestive that, to a first order approximation, the calculation and prediction of just the fragment bearing the “correct” excitation can provide a reasonably accurate prediction.

Upon further analysis of the outliers in these approaches, it is found that the remaining part of the molecule can, in some instances, have a considerable effect. In particular, it was found that most such outliers consist of fragments that were originally part of ring systems, which is consistent with previous studies (for example, a (*Z*)-butene formed from the fragmentation of cyclopentene, for which errors can be as high as 9 kcal mol^−1^).^7^ While this observation suggests that the final predictions could be improved by further refining the fragmentation scheme, an alternative and more robust approach would be the development of a model architecture that can learn the specific contributions of the neighboring environment not encapsulated by the fragment itself.

In light of these results, we attempted to utilize a *Δ*-learning scheme to reduce model error further. Here we keep the same core-fragment prediction scheme detailed above but adapt the architecture and the model training (detailed in Sections S3.1.4 and S3.4 of the SI) to take as inputs both the molecule and the core and output two values: one associated to the adiabatic S_0_–T_1_ gap for the provided core and a second value to account for the contribution of the rest of the molecule. From the SMILES or SMARTS of the core, a mask of the atoms and bonds in the provided core of the molecule is created and used to update two distinct global states iteratively, one for the core (or main fragment) and another for the remaining atoms and bonds of the molecule. From each of these, we obtain a prediction for the core and a correction, whose addition leads to the final S_0_–T_1_ prediction. Historically, *Δ*-learning models have been used to learn error corrections, allowing low-level theory quantum mechanical methods to be matched with results from more accurate computational or experimental methodologies.^49^ This methodology has proven successful in predicting diverse chemical properties, such as solvated ground-state redox potentials,^50^ protein–ligand binding affinities,^51^ and dielectric constants.^52^ Here, we treat our core-fragment prediction model as a “base prediction” and predict a correction to it from the non-core atoms and bonds, thus considering atom contributions from the non-fragment constituent atoms. This adapted model follows a two-stage training process (fully detailed in the SI), where the Fragments-DB is used during the first training stage to train the core-fragment prediction model weights. A second stage then follows, using the combined ALFAST and Fragments-DB to fine-tune the final predictions.

Pleasingly, upon implementation and training of this *Δ*-learning approach, a test set *R*^2^ and MAE of 0.93 and 2.24 kcal mol^−1^ was obtained on the ALFAST-DB test set (Fig. 8E), without a significant degradation in performance over the simplistic augmentation scheme (*R*^2^ = 0.93, MAE = 2.18 kcal mol; Fig. 8B). More intriguingly, however, the results obtained for the last approach are comparable in accuracy to the performance of the GNN trained on and applied to predict exclusively single-fragment molecules (Fig. 5cc and 7). To provide an additional baseline for validation of the predictive power of our developed model, we fine-tuned a large language model (LLM)-based predictor using a 102 million parameter RoBERTa^53^-style model trained on 480 million SMILES strings from the ZINC database.^54^ As the LLM model is trained only to reconstruct SMILES strings, it is agnostic of molecular topology and atom connectivities and thus incorporates no fragment information directly. We find that the fine-tuned LLM model achieves an MAE of 6.17 kcal mol^−1^, which is significantly worse than the task-specific *Δ*-learning approach (MAE 2.24 kcal mol^−1^), highlighting the improvements in accuracy gained by incorporating fragment and molecule graph information. Further details on LLM model training and evaluation can be found in the SI, Section S7 LLM model comparison.

As such, we can conclude that this approach not only removes the excitation localization problem but also can partially account for electronic effects contributions of non-fragment constituent atoms. While further investigations are needed to elucidate the precise quantitative role of the non-fragment constituents, this approach leads to a significant improvement in performance compared to the EnT-decker and our original training approaches, providing a new baseline against which future GNN architectures for predicting excited state properties should be evaluated.

**Fig. 9:**
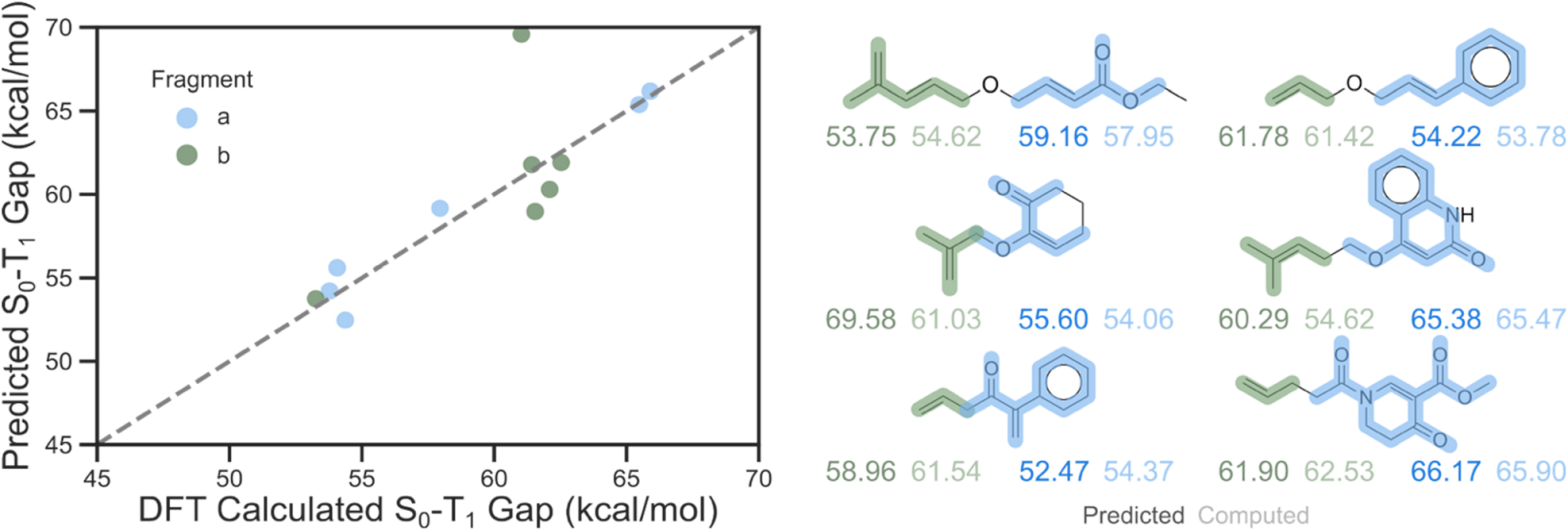
Model validation using a test set of six known molecules that can undergo [2 + 2] cycloaddition upon triplet energy sensitization, together with a parity plot of *Δ*-learning perditions against DFT computed values. Input fragments have been highlighted in green and blue for each molecule.

To demonstrate the applicability of the final *Δ*-learning approach, a test set of six experimentally known molecules that undergo intramolecular [2 + 2] cyclization upon triplet energy sensitization was constructed (Fig. 9).^55–60^ As these transformations inherently require two separated π-systems, two distinct excitations are anticipated, with spin densities localized on either one of the two fragments. Consistent with our expectations, we were able to computationally locate the triplet adiabatic structures for each chromophore at the M06-2X/def2-TZVP level of theory. Using the *Δ*-learning approach with the highlighted fragments as inputs, individual predictions can be provided for each chromophore. Comparison with the DFT-computed results yielded an MAE of 2.09 kcal mol^−1^, which is in good agreement with the model accuracy of 2.24 kcal mol^−1^ reported for the external test set. Notably, only one large outlier was observed for the isobutene fragment, resulting in an error of 8.55 kcal mol^−1^.

## Conclusions

In this work, a fundamental problem in the context of photoredox and energy transfer catalysis is identified: the localization of the exciton. With this problem in mind, we constructed accurate high-throughput computational databases for the development of machine learning models to predict the adiabatic S_0_–T_1_ energy gap for small molecules. To do this, a chemically guided fragmentation algorithm was developed to address database analysis and curation. Next, its application on previously existing databases was showcased, highlighting the impact of exciton delocalization on both previously developed and newly built databases. By analyzing the impact of the fragmentation scheme on the EnT-DB, ALFAST-DB, and Verde-DB datasets, as well as the subsequent changes in performance of GNN models trained on these datasets, principles for designing and augmenting fragment-based datasets targeting the adiabatic S_0_–T_1_ gap were elucidated. To improve database quality, the addition of diverse exciton localization moieties, “main fragments”, is preferable to the addition of duplicated versions of the same moieties, even when such moieties originate in diverse molecular species. Finally, based on these results, a promising strategy for developing ML models to predict the adiabatic S_0_–T_1_ gap was developed, where the prediction of the adiabatic S_0_–T_1_ gap for the key chemical moiety of the target molecule is decoupled from the rest of the molecule. For this strategy to be valid, the property of the molecule must be well approximated by the property of the minimal motif/fragment. We demonstrate here that such a model exhibits a similar error (MAE of 2.3 kcal mol^−1^) to complex machine learning models trained on significantly larger databases (in the range of 1.93–2.26 kcal mol^−1^).

We believe that the present work marks a clear direction for the development of newer fragment-based models and databases in the field, while also highlighting the importance of incorporating domain knowledge into the prediction pipeline and data analysis. Although the final approach with a fragment-based *Δ*-learning model shows good predictive accuracy, we believe further improvements may be made through a better integration or design of the interaction between the core of the molecule and its substituents or the use of even more novel architectures. Future work will take advantage of newly developed GNN architectures that explicitly model this dependence, which also improves model interpretability by allowing explicit fragment contributions to the adiabatic triplet energy to be directly quantified. Furthermore, by incorporating newly released datasets of molecular properties and geometries spanning broad swaths of chemical space calculated using electronic structure methods (such as the OMol25 ^61^ or AIMNet2 ^62^ datasets), the accuracy and generalizability of subsequent models can be greatly improved. While dataset augmentation *via* application-specific quantum chemistry calculations or the use of sophisticated training strategies such as knowledge distillation may need to be employed, the potential for model improvement is substantial, which we will address in our following work.

## Author contributions

Conceptualization, formal analysis, investigation, methodology, validation, visualization, writing – original draft, review & editing: R. P.-S., conceptualization, methodology, formal analysis, investigation, writing – original draft, review & editing: M. V. P., conceptualization, methodology, investigation, writing – original draft, review & editing: Sabari Kumar, methodology, investigation: L. A. G, methodology, investigation: C. L., methodology, writing – review & editing: E. S., conceptualization, formal analysis, writing review & editing: S. A. L., conceptualization, formal analysis, writing review & editing: R. S. P., conceptualization, funding acquisition, project administration, supervision, writing – review & editing: Seonah Kim.

## Conflicts of interest

The authors declare no competing interest.

## Supplementary Material

SC-OLF-D5SC05615B-s001

## Data Availability

Model code and analysis scripts can be found at https://github.com/rperezsoto/aS0T1_prediction_model. Supplementary information: database files as compiled from QM calculations and as used on each of the trainings are available at Zenodo (DOI: https//doi.org/10.5281/zenodo.16563830). See DOI: https://doi.org/10.1039/d5sc05615b.
