# Supplementary material for: A fragment based approach towards curating, comparing and developing machine learning models applied in photochemistry
Source: Chem Sci. 2025 Oct 15;16(46):21874–86. doi: 10.1039/d5sc05615b (PMC12542913; doi:10.1039/d5sc05615b)
Supplement: SC-016-D5SC05615B-s001 [file SC-016-D5SC05615B-s001.pdf]

Supplementary Information (SI) for Chemical Science.
This journal is © The Royal Society of Chemistry 2025

Supporting Information

A Fragment Based Approach Towards Curating, Comparing and Developing Machine Learning Models Applied in Photochemistry

Raúl Pérez-Soto,[a†] Mihai V. Popescu,[a†] Sabari Kumar,[a†] Leticia Adao Gomes,[b] Changyeob Lee,[a] Elijah Shore,[a] Steven A. Lopez,[b*] Rob S. Paton,[a*] and Seonah Kim[a*]
[a] Department of Chemistry, Colorado State University, Fort Collins, Colorado 80523, USA.
[b] Department of Chemistry and Chemical Biology, Northeastern University, Boston, Massachusetts, USA.

S1. Database Generation: Computational Details 2
S1.1. ALFAST-DB and Fragments-DB 2
S1.2. Verde-DB Extension 2
S2. Metrics and cross-validation schemes 3
S3. Architecture Selection 3
S3.1. Candidate architectures 3
S3.1.1 Non-standard Layers and Operations Description 3
S3.1.2 General Architecture 5
S3.1.3 Architectures 6
S3.1.4 Delta Learning Model Architecture 6
S3.2. Hyperparameter tuning 8
S3.3. Final Model Metrics 13
S3.4. Delta Learning Model Details 14
S4. Fragmentation analysis extended 14
S4.1. Database Comparison Details 14
S4.2. Main Spin Labeling: ALFAST-DB and Fragments-DB 15
S5. Additional Figures of database contents 15
S6. LLM Model Comparison 19
S7. XYZ coordinates 20
S8. References 34

S1. Database Generation: Computational Details

S1.1. ALFAST-DB and Fragments-DB

We screened the database ALFABET only for neutral molecules containing a double, triple or aromatic bond, through the usage of SMARTS and the pybel library of openbabel,[ref1] leading to a total of 57 736 molecules. As initial geometries for the triplet optimization we started from the already optimized singlet geometries (from ALFABET) and re-optimized the molecules in the triplet energy surface using Gaussian 16 at the M06-2X/def2-TZVP level with the nosymm keyword. Initially this led to a total of 39 225 calculations that converged to a minima while most of the remaining molecules failed either due to convergence during the optimization or reached a stationary point with an imaginary frequency. To automate the check of the calculation termination and to extract the geometries, atomic Mulliken spins we used the pyssian library.[ref2]

Then, we performed a second round of calculations. For this round we distorted the molecules with imaginary frequencies along the imaginary mode (the full distortion in the positive direction as determined by Gaussian). For molecules that failed at the frequency calculation stage the last geometry was used and for the other failed calculations the geometry with the lowest potential energy throughout the optimization was used. After the second round of calculations a total of 53 137 calculations had converged.

We then proceeded to screen the converged structures to ensure that the connectivity of the molecules remained the same (further detailed in the SI) which lowered the total number of molecules to the final ALFAST-DB size, 46 415 molecules. This final geometry screen was carried out using, the openbabel and the networkx libraries. First, through openbabel's conversion from xyz format to SMILES the geometry was checked to ensure that the molecules did not dissociate into 2 different molecules during the optimization (presence of a "." in the SMILES) and the canonicalized SMILES in the database and the canonicalized SMILES of the xyz-to-SMILES conversion were tested for equality. As in some cases small variations in the bond-distance could lead to changes in the SMILES, two graph isomorphism checks were carried out using networkx. The first one, where only categorical node matches (atoms) were considered in the graph isomorphism between the graph of the initial SMILES and the graph of the SMILES obtained from the xyz-to-SMILES conversion. Then a more restrictive graph isomorphism with categorical matches for edges and nodes was considered, which provided similar results to a canonical SMILES comparison. Thus, we only removed molecules whose graph isomorphism with categorical node matching found no match between the initial SMILES and the SMILES perceived from the optimized geometry. The categories of the nodes were the atomic number of the atom it represented.

For the Fragments-DB due to the characteristics of this database ("monocore" small molecules with a restricted conformational variability) the initial geometries were generated from SMILES using openbabel's distance geometry implementation and initially relaxed using Conjugated Gradients with a stopping criteria of 350 steps or 1E-4 energy difference using MMFF94 when possible and for the molecules that failed the UFF forcefield was used instead. From these starting geometries a GFN2-XTB optimization with verytight optimization criteria was carried out using XTB 6.7.1 [ref3] for both, the singlet and the triplet. The obtained triplet geometries were pre-screened using the same categorical node matching graph isomorphism check used for ALFAST-DB. Molecules that were not discarded were then used for the optimization at the M06-2X/def2-TZVP level using Gaussian 16 (respectively using the xtb-optimized singlet geometry as initial geometry for the singlet DFT optimization and the xtb-optimized triplet geometry for the triplet DFT optimization). At this stage, the same procedure as with ALFAST-DB was carried out.

Unfortunately, at a later stage in the study we found that a small subset of molecules that had undergone an H-transfer during the optimization were included in the curated dataset. We found that it is due to the initial SMILES not including explicit H atoms, therefore its graph representation would not have H-atoms and would be treated as a subgraph of the graph obtained from the optimized geometry. As these amount to a very small quantity of compounds (all corresponding a subset of the molecules with a main fragment with a total Mulliken of ~1, see Figure 4a in the manuscript) relative to the database size we did not repeat the full study as we predicted them to have a low impact on the model performance.

S1.2. Verde-DB Extension

Our objective was to broaden the scope of our database by including light-responsive π-conjugated organic molecules with potential applications in organic redox flow batteries. To achieve this, we integrated new core structures including quinoline-5,8-dione and quinoxaline-5,8-dione, which feature systematic functionalization at each open

site. We utilized an in-house algorithm that systematically links 20 spacer and 11 terminal groups to generate the virtual library in line with the VERDE materials DB.

S2. Metrics and cross-validation schemes

To evaluate the performance of the models on unseen data we used a 80:20 train-validation:test split and we used a five-fold cross validation to select the optimal hyperparameters of each MPGNN architecture explored (ultimately being a training:validation ratio of 64:16, relative to the full dataset, per each fold). To ensure that the comparison of the results of each fold across the different models we decided to always use the same fold partitions. The splits of the combined ALFAST-DB and Fragments-DB were the combination of the respective splits of each database.

The metrics used to evaluate the performance of the models were the Mean Absolute Error (MAE), and the R^2 correlation coefficient between the predictions and the true values. These metrics were used as implemented in the tensorflow library and the general equations are shown below, where n is the number of samples y_{true} and y_{pred} are the true and predicted values of each sample.

$$1)\ MAE = \frac{\sum_i^n \left|y_{i,true} - y_{i,pred}\right|}{n}$$

$$2)\ R^2 = 1 - \frac{\sum_i^n \left(y_{i,true} - y_{i,pred}\right)^2}{\sum_i^n \left(y_{i,true} - \overline{y_{true}}\right)^2}$$

When cross-validation results are provided, they are accumulated across folds. For example, in the case of the MAE, per each fold's validation set the predictions and the absolute errors are determined using only the model trained with the fold's training set, then the total absolute error per fold is added across folds and finally divided by the size of the dataset, mathematically:

$$3)\ MAE_{CV} = \frac{\sum_{k=0}^{folds} \sum_i^{n_k} \left|y_{i,true} - y_{i,pred}\right|}{n}$$

Finally, when test set metrics are provided they are the mean of the metric across different folds:

$$4)\ \overline{MAE_{Test}} = \frac{\sum_k^{folds} MAE_{Test,k}}{folds}$$

S3. Architecture Selection

S3.1. Candidate architectures

S3.1.1 Non-standard Layers and Operations Description

Dense Concatenation Layer: this layer is a composite layer (nfp.ConcatDense) that adds more weights to the model. First a concatenation in the axis of the number of features is carried out (which involves no new weights). As the nodes, bonds and global state are always considered the same size (embedding dimension), at least it duplicates the number of features. The outcome of the concatenation is fed to a Dense layer of size "2 x embedding dimension" with a ReLU activation and bias, whose outcome is then fed to a Dense layer of the same size of the embedding dimension with a lineal activation (in other words, no activation function) and bias. These last two Dense layers are the ones actually including new weights into the model.

Reduce Layer: This layer is a composite layer (nfp.Reduce) involving a reduction operation (in our case always a summation) that does not add any new weights to the model. It acts as a wrapper to allow the reduction of the messages for each node, without a self-update of each node, which has to be done afterwards.

Node Update: a node update operation (nfp.NodeUpdate) is a sequence of operations that overall updates the node states. First, a gather (nfp.Gather) layer is used to gather the end nodes of each edge in the graph, which are the sources of the messages. Edges in the graph in this context are always directional, and each bond is represented by two edges, one per direction. Next, a Dense Concatenation Layer between the edge states and the gathered end nodes is included, obtaining a message per edge. These are fed to a Reduce Layer coupled with the node states and the connectivity to aggregate the messages that each node receives. The aggregated messages are then fed sequentially to a Dense layer of size "2 x embedding dimension" with a ReLU activation and bias and then to a Dense layer with a lineal activation and bias obtaining a final form of the messages. These messages which are of the same dimensions as the node states are added to the node states to account for the self-update of the node. The outcome of all these steps is the updated state of the nodes. When a Global state is also passed as input, it is tiled to match the dimensions and also fed to the Dense Concatenation Layer.

Bond Update: a bond update operation (nfp.BondUpdate) is a sequence of operations that overall updates the bond states. First a gather (nfp.Gather) operation is used to gather the start nodes of each edge as well as the end nodes of each edge, which are then subsequently fed to a Dense Concatenation Layer with the bond state, whose outcome is then added to the bond state to obtain the updated bond state. When a Global state is also passed as input, it is tiled to match the dimensions and also fed to the Dense Concatenation Layer.

Global Update: When no previous Global state is defined, this operation pools the information from the nodes and edges of the graph generating an initial graph embedding of the same dimension as the bonds or nodes. When used to update the Global state, as with the Node Update or Bond Update, a final step involving a self-update through an addition operation is carried out. In this operation (nfp.GlobalUpdate) an attention mechanism is built into the pooling from the graph elements, thus the number of attention heads and the number of units needs to be specified and its product should match the embedding dimension used for the bonds and the atoms. We specifically used 1 attention head, and therefore the number of units and the embedding dimension are equal. First, in this operation nodes and bond states are concatenated (note that this is not a Dense Concatenation, but a concatenation across the dimension corresponding to the number of atoms and number of bonds instead of concatenating along the axis of the features). The outcome of this operation is the Graph Element state tensor, which is first fed to a query layer (a Dense layer of size 1 with bias and no activation function) and subsequently subjected to a softmax operation generating the attentions. Next, the Graph Element state tensor is also fed to a value layer (a Dense layer of the size of the embedding dimension with bias and no activation function) generating the values. The matrix multiplication of the values and the transposed attentions generates the final weighted graph element values are added together generating a global state update vector of the same dimension as the embedding dimension. Finally, if this operation is used to update a previously existing global state, an addition operation is carried out.

Masked Global Update: Exactly like Global Update but it takes as input also a list of node indices and edge indices with which to mask the attentions to ensure that only those nodes and edges contribute to the new global state. This is not an existing layer in the nfp package and the implementation is available with all the code associated to the present work available in our github.

Message Passing operation: A message passing operation, here, involves a Bond Update, Node Update and Global Update in this specific order to update the previous bond states, node states and global state.

S3.1.2 General Architecture

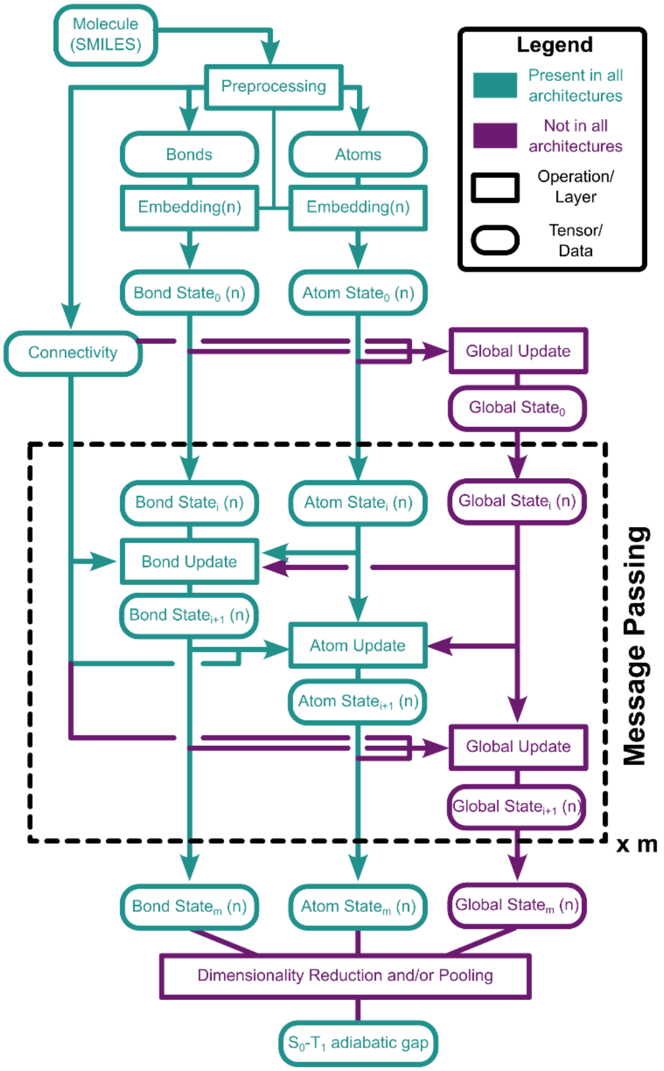

Figure S1: Diagram illustrating the common part of all the Message Passing Graph Neural Network architectures explored in the present work. "n" stands for the embedding dimension and "m" for the number of message-passing iterations.

A depiction of the general message passing architecture is used as the base for the explored model variations is shown above (Figure S1). The preprocessing used was the same across the architectures. It was implemented using the nfp SmilesBondIndexPreprocessor and the atom and bond features used for the preprocessor were:

- Atom features
  - Atom Symbol.
  - Number of Radical Electrons.
  - Formal Charge.
  - Chiral Tag.
  - Whether it is an aromatic atom or not.
  - The size of the smallest ring it belongs to.
  - Degree (Number of directly bonded neighbors)
  - The total number of H atoms bonded to.
- Bond features (each bond is represented by two directed edges one starting on each atom involved in the bond)
  - Atomic Symbol of the starting atom
  - Atomic Symbol of the end atom
  - Type of bond (single, aromatic, double or triple)
  - Whether it belongs to a ring or not.
  - Smallest ring the bond belongs to.

S3.1.3 Architectures

Architecture diagrams of the pooling and dimensionality reduction sections for models detailed in section S3.2 are provided below. “n” stands for the embedding dimension and “m” for the number of message passing iterations:

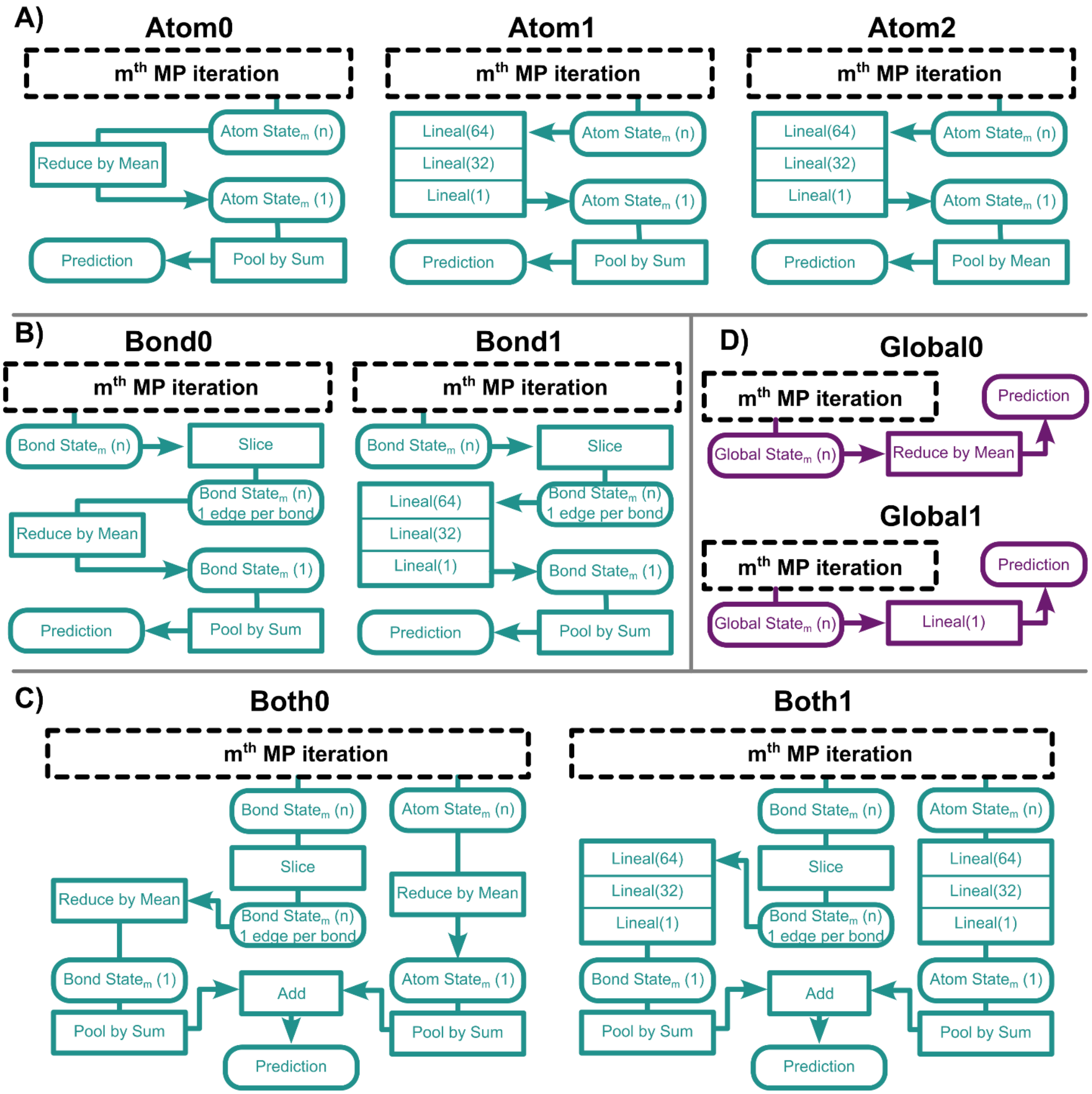

Figure S2: A) Atom pooling architectures. Predictions are generated by pooling node feature representations.B) Bond pooling architectures. Predictions are generated by pooling edge feature representations. C) "Both" pooling architectures. Predictions are generated using a sum of pooled node and edge representations. D) Global pooling architectures. Predictions are generated by doing a dimensionality reduction of the global states which pool the atoms and bonds data during the Message Passing iterations. MP stands for message passing. The number between brackets correspond to the output dimension. The same legend of Figure S1 applies.

S3.1.4 Delta Learning Model Architecture

The proposed delta learning model architecture and training was adapted from the Global1 architecture. Its architecture is almost identical, except for the presence of two extra global states, which are updated in parallel to the

global state (See Figure S6). In this manner we have the original global state, to represent the full molecule and to interact in the updates of edges and nodes. This global state is always updated with a Global Update layer (nfp.GlobalUpdate). The two extra global states, one to represent the "core" (exciton location) and one to represent the "noncore" (remaining set of atoms and bonds) are instead updated using a Masked Global Update. After the message passing layers, the "core" and "noncore" states are fed each to a Lineal layer of size 1 to obtain respectively the "core energy gap" and the "noncore energy correction". These two values are finally added together to obtain the final prediction. Further details on the custom training of this model are presented in the Delta Learning Model Details.

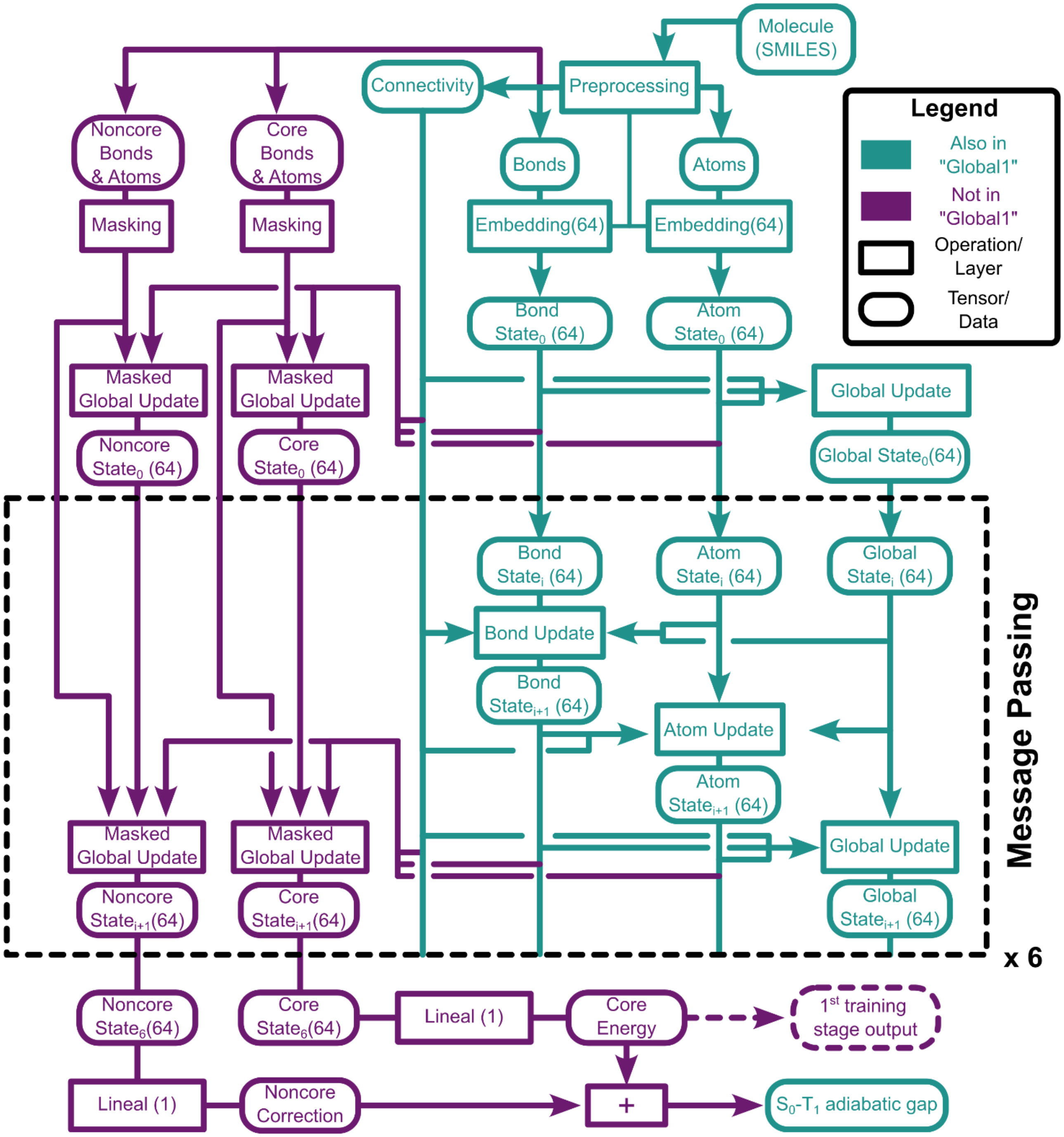

Figure S3: Diagram illustrating the final delta-fragment model architecture used in the present work. Numbers between brackets stand the embedding dimension used or output dimension of an operation/layer.

The preprocessor used for the training of this architecture is derived from nfp SmilesPreprocessor and was adapted to take as input the SMILES of the target molecule and the SMILES or SMARTS of the core as inputs. As for the target molecule, this preprocessor also builds a graph for the core and after obtaining the atom indices of the target molecule that match the core it finds a atom and bond mapping across both graphs and extracts a list of node indices and edge indices in the core as well as another list of node indices and edge indices for the atoms not present in the

core. A special argument "full" may be passed as core, to ensure that all atoms and bonds will be considered as part of the core. This special argument was included to simplify the first stage of the training.

S3.2. Hyperparameter tuning

The results of hyperparameter optimization are shown below. Selected hyperparameters for the final model are highlighted. All optimizations were carried out using an AdamW optimizer with an Inverse Time Decay learning rate scheduler and a constant weight decay rate. The batchsize was kept constant at 128 (molecules per batch). In all cases the loss function as the MAE. During model training the validation loss was tracked and the model weights with the best validation loss were also saved, and the values of such model are shown as "Best Loss" in Tables S1 and S2.

Table S1: Architecture selection. The initial learning rate was kept constant at 1E-3, the learning rate decay was kept at 1E-5 and the weight decay was kept at 1E-5.

			5-fold mean values, kcal/mol			
			Final Loss (epoch 450)		Best Loss	
**Architecture**	**MP Layers**	**Embedding Dimension**	**Training**	**Validation**	**Training**	**Validation**
atom0	2	16	2,85	3,53	2,89	3,42
		32	2,23	3,27	2,23	3,15
		64	1,76	3,10	1,79	3,02
		128	1,65	3,16	1,68	3,07
	3	16	2,25	3,05	2,25	3,00
		32	1,46	2,62	1,49	2,58
		64	1,12	2,62	1,11	2,50
		128	1,01	2,61	1,06	2,54
	4	16	1,90	2,80	1,94	2,74
		32	1,27	2,58	1,31	2,48
		64	1,00	2,48	1,03	2,42
		128	0,94	2,56	0,98	2,43
	5	16	1,62	2,67	1,66	2,55
		32	1,16	2,44	1,20	2,35
		64	0,88	2,35	0,93	2,32
		128	0,96	2,49	0,99	2,43
	6	16	1,56	2,67	1,60	2,52
		32	1,05	2,37	1,06	2,25
		64	0,83	2,30	0,88	2,25
		128	0,93	2,44	0,99	2,28
atom1	2	16	2,48	3,26	2,49	3,17
		32	1,91	3,10	1,94	2,94
		64	1,60	2,98	1,65	2,92
		128	1,56	3,02	1,60	2,97
	3	16	1,92	2,80	1,95	2,70
		32	1,37	2,53	1,42	2,46
		64	1,02	2,46	1,07	2,38
		128	1,04	2,59	1,09	2,52
	4	16	1,67	2,71	1,74	2,56
		32	1,17	2,45	1,21	2,33
		64	0,89	2,36	0,95	2,27
		128	0,90	2,44	0,93	2,37
	5	16	1,57	2,64	1,63	2,49
		32	1,05	2,37	1,10	2,23
		64	0,80	2,26	0,90	2,19

		128	0,84	2,29	0,87	2,25
	6	16	1,46	2,48	1,47	2,40
		32	0,97	2,25	1,00	2,18
		64	0,79	2,27	0,81	2,14
		128	0,79	2,27	0,80	2,17
atom2	2	16	2,50	3,23	2,51	3,03
		32	2,02	2,92	2,06	2,82
		64	1,71	2,92	1,84	2,76
		128	1,62	2,88	1,63	2,81
	3	16	1,93	2,61	1,95	2,53
		32	1,43	2,42	1,53	2,35
		64	1,16	2,31	1,18	2,26
		128	1,06	2,38	1,23	2,27
	4	16	1,75	2,52	1,76	2,38
		32	1,31	2,34	1,34	2,24
		64	1,08	2,30	1,15	2,18
		128	1,03	2,34	1,08	2,18
	5	16	1,64	2,46	1,66	2,34
		32	1,26	2,26	1,32	2,20
		64	1,04	2,23	1,10	2,12
		128	1,02	2,29	1,08	2,13
	6	16	1,58	2,43	1,61	2,31
		32	1,19	2,28	1,25	2,17
		64	1,01	2,17	1,09	2,09
		128	1,01	2,25	1,02	2,10
bond0	2	16	4,95	5,82	4,92	5,69
		32	4,29	5,34	4,29	5,21
		64	3,72	4,90	3,69	4,84
		128	3,44	5,07	3,49	4,82
	3	16	3,85	4,78	3,82	4,62
		32	2,84	4,13	2,89	3,92
		64	2,11	3,70	2,10	3,49
		128	2,05	3,60	2,09	3,50
	4	16	3,41	4,43	3,44	4,29
		32	2,43	3,69	2,45	3,59
		64	1,81	3,37	1,79	3,19
		128	1,74	3,26	1,70	3,21
	5	16	3,19	4,26	3,14	4,07
		32	2,19	3,59	2,17	3,42
		64	1,67	3,16	1,67	3,10
		128	1,62	3,36	1,65	3,15
	6	16	2,83	3,91	2,88	3,86
		32	1,99	3,41	2,00	3,27
		64	1,57	3,20	1,59	3,07
		128	1,62	3,20	1,64	3,12
bond1	2	16	4,64	5,48	4,64	5,42
		32	4,15	5,23	4,10	5,07
		64	3,59	5,09	3,63	4,91
		128	3,58	5,01	3,61	4,97
	3	16	3,49	4,79	3,56	4,35

		32	2,60	3,94	2,55	3,71
		64	2,11	3,73	2,10	3,52
		128	2,41	4,02	2,43	3,84
	4	16	3,17	4,10	3,18	4,04
		32	2,10	3,38	2,18	3,35
		64	1,77	3,25	1,78	3,18
		128	2,04	3,52	2,07	3,46
	5	16	2,89	3,89	2,91	3,80
		32	1,94	3,41	1,96	3,20
		64	1,70	3,10	1,67	3,01
		128	1,95	3,44	2,02	3,32
	6	16	2,71	3,83	2,70	3,65
		32	1,78	3,14	1,73	2,94
		64	1,49	3,14	1,51	2,86
		128	1,94	3,40	1,98	3,28
both0	2	16	3,16	3,93	3,17	3,77
		32	2,31	3,52	2,34	3,31
		64	1,86	3,24	1,89	3,21
		128	1,95	3,47	1,95	3,41
	3	16	2,28	3,15	2,29	3,07
		32	1,46	2,74	1,51	2,66
		64	1,18	2,73	1,20	2,66
		128	1,19	2,87	1,25	2,80
	4	16	1,96	2,99	1,92	2,76
		32	1,30	2,74	1,32	2,57
		64	0,97	2,63	0,99	2,53
		128	0,99	2,70	1,00	2,60
	5	16	1,74	2,67	1,77	2,65
		32	1,15	2,67	1,19	2,40
		64	0,93	2,58	0,94	2,48
		128	0,98	2,59	1,01	2,51
	6	16	1,59	2,69	1,61	2,54
		32	1,06	2,44	1,11	2,36
		64	0,88	2,43	0,91	2,35
		128	0,99	2,59	1,05	2,48
both1	2	16	2,65	3,41	2,65	3,32
		32	1,97	3,06	2,00	2,98
		64	1,74	3,11	1,72	2,99
		128	1,74	3,38	1,76	3,15
	3	16	1,94	2,94	1,99	2,75
		32	1,39	2,59	1,42	2,47
		64	1,06	2,48	1,07	2,41
		128	1,08	2,59	1,11	2,49
	4	16	1,69	2,61	1,68	2,53
		32	1,20	2,45	1,22	2,36
		64	0,91	2,38	0,94	2,26
		128	0,93	2,43	0,93	2,32
	5	16	1,58	2,63	1,59	2,50
		32	1,08	2,43	1,09	2,27
		64	0,85	2,23	0,86	2,20

		128	0,86	2,39	0,88	2,24
	6	16	1,48	2,55	1,51	2,41
		32	1,00	2,25	1,07	2,19
		64	0,79	2,27	0,83	2,14
		128	0,84	2,25	0,87	2,18
global0	2	16	1,94	2,59	1,98	2,49
		32	1,38	2,36	1,41	2,26
		64	1,02	2,43	1,05	2,22
		128	0,84	2,29	0,92	2,18
	3	16	1,63	2,40	1,66	2,28
		32	1,11	2,18	1,20	2,08
		64	0,77	2,08	0,84	2,04
		128	0,63	2,17	0,67	2,00
	4	16	1,49	2,34	1,50	2,14
		32	0,98	2,10	1,06	2,03
		64	0,66	2,03	0,74	1,93
		128	0,61	1,99	0,65	1,89
	5	16	1,39	2,16	1,46	2,09
		32	0,86	2,08	0,94	1,94
		64	0,58	1,95	0,63	1,88
		128	0,54	1,98	0,58	1,84
	6	16	1,29	2,14	1,33	2,06
		32	0,79	2,04	0,82	1,92
		64	0,55	1,93	0,66	1,83
		128	0,53	1,89	0,56	1,82
global1	2	16	1,64	2,37	1,66	2,26
		32	1,11	2,18	1,19	2,10
		64	0,74	2,05	0,92	2,00
		128	0,64	2,04	0,65	1,97
	3	16	1,48	2,24	1,53	2,13
		32	0,96	2,14	1,11	2,00
		64	0,65	2,04	0,72	1,90
		128	0,53	1,90	0,58	1,86
	4	16	1,39	2,34	1,43	2,09
		32	0,80	2,08	0,86	1,92
		64	0,54	1,88	0,58	1,83
		128	0,50	1,83	0,53	1,79
	5	16	1,25	2,08	1,29	2,03
		32	0,71	1,96	0,81	1,89
		64	0,52	1,83	0,59	1,79
		128	0,46	1,81	0,48	1,75
	6	16	1,15	2,13	1,22	2,01
		32	0,70	1,88	0,80	1,83
		64	0,48	1,79	0,58	1,76
		128	0,46	1,82	0,48	1,73

Table S2: Learning Rate optimization table. Embedding dimension was kept constant at 128 and the Global1 architecture was used.

					5-fold mean values, kcal/mol			
					Final Loss (Last epoch)		Best Loss	
**MP Layers**	**Epochs**	**Learning Rate**	**Learning Rate Decay**	**Weight Decay**	**Training**	**Validation**	**Training**	**Validation**
2	1000	0,00001	0,00001	0,000001	2,09	2,61	2,09	2,55
				0,00001	3,54	3,73	3,33	3,43
				0,0001	6,86	6,95	4,80	4,86
	450	0,00001	0,00001	0,000001	2,31	2,76	2,31	2,72
				0,00001	3,41	3,58	3,39	3,48
				0,0001	5,44	5,57	5,06	5,11
		0,0001	0,00001	0,000001	0,83	2,15	0,98	2,12
				0,00001	1,30	2,24	1,38	2,17
				0,0001	3,12	3,43	3,11	3,20
			0,1	0,000001	10,62	10,65	10,32	10,35
				0,00001	72,89	72,89	11,48	11,48
				0,0001	72,97	72,97	13,68	13,66
			0,3	0,000001	28,12	28,17	14,95	14,94
				0,00001	72,94	72,94	37,32	37,28
				0,0001	72,97	72,97	67,79	67,76
		0,001	0,00001	0,000001	0,53	2,08	0,61	1,99
				0,00001	0,63	2,08	0,67	1,97
				0,0001	1,50	2,43	1,54	2,09
			0,1	0,000001	4,66	4,74	4,66	4,74
				0,00001	8,75	8,79	5,07	5,13
				0,0001	72,97	72,97	6,63	6,68
			0,3	0,000001	6,81	6,86	6,77	6,82
				0,00001	71,85	71,86	7,73	7,76
				0,0001	72,97	72,97	8,75	8,76
6	1000	0,00001	0,00001	0,000001	1,25	2,31	1,30	2,23
				0,00001	2,81	3,02	2,51	2,72
				0,0001	5,82	5,94	5,16	5,20
	450	0,00001	0,00001	0,000001	1,36	2,31	1,39	2,22
				0,00001	2,61	3,10	2,48	2,71
				0,0001	5,26	5,35	5,15	5,19
		0,0001	0,00001	0,000001	0,36	1,84	0,46	1,79
				0,00001	0,74	1,97	0,84	1,86
				0,0001	2,41	2,84	2,40	2,38
			0,1	0,000001	7,92	7,97	6,74	6,82
				0,00001	72,85	72,85	8,42	8,47
				0,0001	72,97	72,97	9,87	9,81
			0,3	0,000001	18,24	18,31	9,94	9,97
				0,00001	72,93	72,93	10,55	10,56
				0,0001	72,97	72,97	12,46	12,38
		0,001	0,00001	0,000001	0,42	1,80	0,45	1,75
				0,00001	0,45	1,77	0,45	1,72
				0,0001	1,26	2,19	1,27	1,89
			0,1	0,000001	3,25	3,41	3,16	3,34
				0,00001	8,03	8,07	3,98	4,07
				0,0001	72,97	72,97	5,34	5,37
			0,3	0,000001	4,71	4,79	4,55	4,64
				0,00001	69,10	69,14	5,81	5,86
				0,0001	72,97	72,97	7,72	7,71

S3.3. Final Model Metrics

The computed metrics of the selected architecture across the different datasets shown in the manuscript are presented below. The trainings followed the same protocol as the one indicated in S3.2. The presented results correspond to the "best" model weights during training, determined as the ones with the lowest validation loss value. In the case of the "final" models, the weights that produced the lowest loss value during training were selected. Here by "final" models we refer to the selected architecture trained with the combined training and validation data splits as training set of each database respectively while still keeping the test set data unseen during training (and only used for evaluation after the training).

Table S3: Computed metrics of the selected architecture with the chosen hyperparameters. Folds 0-4 are the individual results of each one of the individual training:validation folds. CV entries contain the averaged value across folds except for the validation set (*) which are the aggregated metrics instead of the averaged ones. The "final" entries are the models trained using the training and validation as training set, therefore no validation metrics were computed.

MAE in kcal/mol		Training Set		Validation Set		Test Set	
Dataset	CV Fold	MAE	R^2	MAE	R^2	MAE	R^2
alfast	0	1,24	0,9726	1,97	0,9350	2,02	0,9275
	1	1,17	0,9744	1,95	0,9324	1,98	0,9274
	2	1,20	0,9718	1,92	0,9340	1,98	0,9277
	3	1,33	0,9685	1,94	0,9346	2,03	0,9265
	4	1,17	0,9747	1,90	0,9340	2,00	0,9268
	CV	1,22	0,9724	1,94*	0,9340*	2,00	0,9272
alfast	final	1,25	0,9682	--	--	1,94	0,9280
ent	0	0,96	0,9875	2,02	0,9303	2,03	0,9330
	1	0,89	0,9890	2,06	0,9261	2,08	0,9297
	2	1,14	0,9817	2,04	0,9323	2,05	0,9339
	3	1,15	0,9818	2,02	0,9358	2,07	0,9292
	4	1,04	0,9847	2,06	0,9314	2,07	0,9310
	CV	1,04	0,9849	2,04*	0,9312*	2,06	0,9314
ent	final	1,50	0,9774	--	--	2,37	0,9205
verde	0	0,84	0,9765	1,17	0,9548	1,20	0,9590
	1	0,63	0,9886	1,12	0,9684	1,12	0,9644
	2	0,85	0,9757	1,16	0,9578	1,13	0,9611
	3	0,70	0,9860	1,07	0,9586	1,11	0,9642
	4	0,44	0,9946	1,18	0,9608	1,16	0,9599
	CV	0,69	0,9843	1,14*	0,9604*	1,14	0,9617
verde	final	0,45	0,9950	--	--	1,27	0,9564
fragments	0	0,95	0,9783	2,68	0,8502	3,05	0,8287
	1	1,27	0,9579	2,98	0,8283	3,30	0,8048
	2	1,29	0,9600	3,34	0,7811	3,30	0,8008
	3	1,39	0,9453	3,29	0,7977	3,38	0,7759
	4	1,31	0,9569	3,07	0,8361	3,27	0,7987
	CV	1,24	0,9597	3,07*	0,8180*	3,26	0,8018
fragments	final	0,90	0,9813	--	--	2,98	0,8309
alfast-fragments	0	1,29	0,9664	1,92	0,9321	1,99	0,9246
	1	1,16	0,9730	1,92	0,9301	1,99	0,9272
	2	1,09	0,9775	1,91	0,9347	1,95	0,9293
	3	1,22	0,9692	1,94	0,9318	2,00	0,9241
	4	1,25	0,9721	1,93	0,9384	2,04	0,9265
	CV	1,20	0,9716	1,92*	0,9334*	1,99	0,9263
alfast-fragments	final	1,53	0,9674	--	--	2,21	0,9254

S3.4. Delta Learning Model Details

The training of this architecture, is also adapted from the training of the Global1 architecture. A two stage training is carried out. In the first stage of the training all weights associated to the noncore global state are frozen, and the "core energy" is taken as the model output and directly compared with the training S_0-T_1 adiabatic gap values of the Fragments-DB (see Figure S3). In the second stage of the training all weights are allowed to optimized and the outcome of "core energy" + "noncore correction" is taken as the model output and compared again, against the S_0-T_1 adiabatic gap values.

- **Stage 1:** Core training
  - **Preprocessor:** trained on training subset of the combined ALFAST-Fragments DB
  - **Training Dataset:** Fragments-DB training and validation sets used during training. The preprocessing is tweaked to ensure that all atoms, including H, are labeled as being part of the core.
  - **Optimizer:** AdamW
    - Inverse Time Decay Scheduler
      - Initial LR: 1E-3
      - LR Decay: 1E-5
      - Weight Decay: 1E-4
  - **Loss:** Mean Absolute Error
  - **Epochs:** 200
  - **Batch size:** 128
- **Stage 2:** Delta learning
  - **Preprocessor:** Same preprocessor
  - **Training Dataset:** The combined ALFAST-DB and Fragments-DB training and validation sets. The cores provided to the model in the case of ALFAST are the SMARTS of the main fragments while for Fragments are the full SMILES of the molecule (which is a valid SMARTS)
  - **Optimizer:** AdamW
    - Inverse Time Decay Scheduler
      - Initial LR: 1E-3
      - LR Decay: 1E-5
      - Weight Decay: 1E-4
  - **Loss:** Mean Absolute Error
  - **Epochs:** 450
  - **Batch size:** 128

Table S4: Computed metrics of the delta-learning based model. Folds 0-4 are the individual results of each one of the individual training:validation folds. CV entries contain the averaged value across folds except for the validation set (*) which are the aggregated metrics instead of the averaged ones. The "final" entries are the models trained using the training and validation as training set, therefore no validation metrics were computed.

MAE in kcal/mol		Training Set		Validation Set		Test Set	
Dataset	CV Fold	MAE	R^2	MAE	R^2	MAE	R^2
alfast-fragments	0	1,15	0,9743	1,90	0,9358	1,95	0,9275
	1	1,16	0,9764	1,96	0,9334	2,01	0,9274
	2	1,14	0,9748	1,90	0,9339	1,95	0,9277
	3	1,16	0,9766	1,97	0,9296	1,93	0,9265
	4	1,49	0,9720	2,19	0,9313	2,26	0,9268
	CV	1,22	0,9748	1,98*	0,9328*	2,02	0,9280
alfast-fragments	final	1,67	0,9639	--	--	2,26	0,9256

S4. Fragmentation analysis extended

S4.1. Database Comparison Details

Comparison of the chemical space overlap between EnT-DB and ALFAST-DB was performed via a comparison of the SMILES string representations of the constituent molecules. Constituent SMILES were canonicalized using rdkit's MolToSmiles and MolFromSmiles functions. Then, an inner join of both database sets on the canonicalized

SMILES strings was performed using Python. Figure S7 was obtained from the subset of common molecules determined through this procedure.

When fragment overlap analysis was carried out, each molecule was fragmented using the algorithm described in the main text, translated into SMILES and any duplicate of these fragments was discarded. Then the overlap between the unique pool of fragments of each dataset was assessed following the same procedure as for molecules.

S4.2. Main Spin Labeling: ALFAST-DB and Fragments-DB

For the molecules where we have the atomic Mulliken spin density at the triplet state (specifically, molecules in ALFAST-DB and Fragments-DB), the steps 1 and 2 of the fragmentation algorithm described in Section "Molecular Fragmentation Algorithm" (Figure 2 in the main text), were carried out and followed by adding together the atomic Mulliken spin density of each atom in the fragment, and the one with the highest value of total Mulliken spin was assigned as the moiety where the triplet is mostly localized.
Finally, in the provided ALFAST-DB the remaining unassigned molecules were labeled and assigned based on the smallest difference between the predicted S_0-T_1 of each fragment and DFT value of the molecule. This "empirical" labeling was not used in the present work, and an additional column is provided with the dataset to allow researchers using the provided data to be able to differentiate between correct assignments and proposed ("empirical") assignments.

S5. Additional Figures of database contents

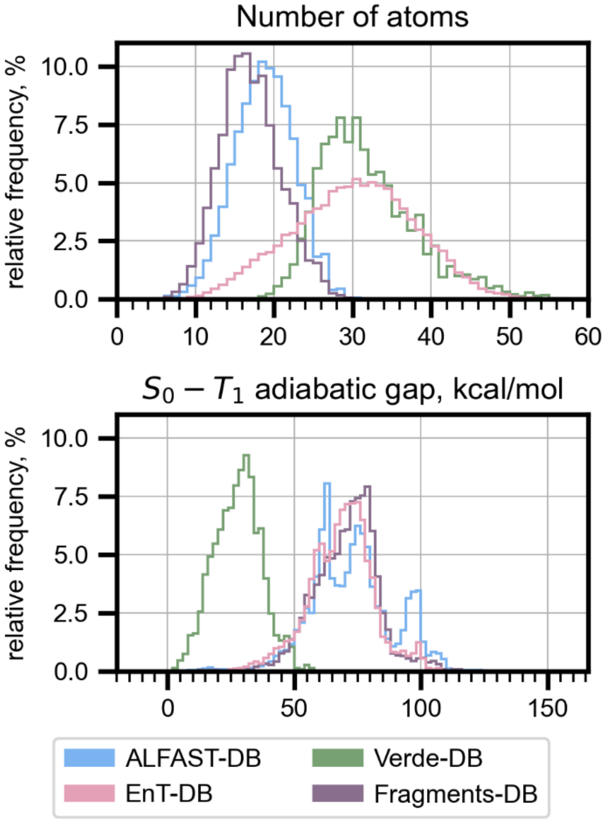

Figure S4: Total number of atoms and computed S_0-T_1 adiabatic gap distributions of the 4 discussed datasets. The bin size selected is of 1 kcal/mol for the S_0-T_1 adiabatic gap and of 1 atom for the number of atoms.

Table S5: Number of shared fragments between databases. The diagonal terms correspond to the total number of fragments of each database.

Databases	ALFAST	EnT	Verde	Fragments
**ALFAST**	15 410	2 146	0	3 764
**EnT**	2 146	18 909	0	3 659
**Verde**	0	0	3 286	0
**Fragments**	3 764	3 659	0	5 315

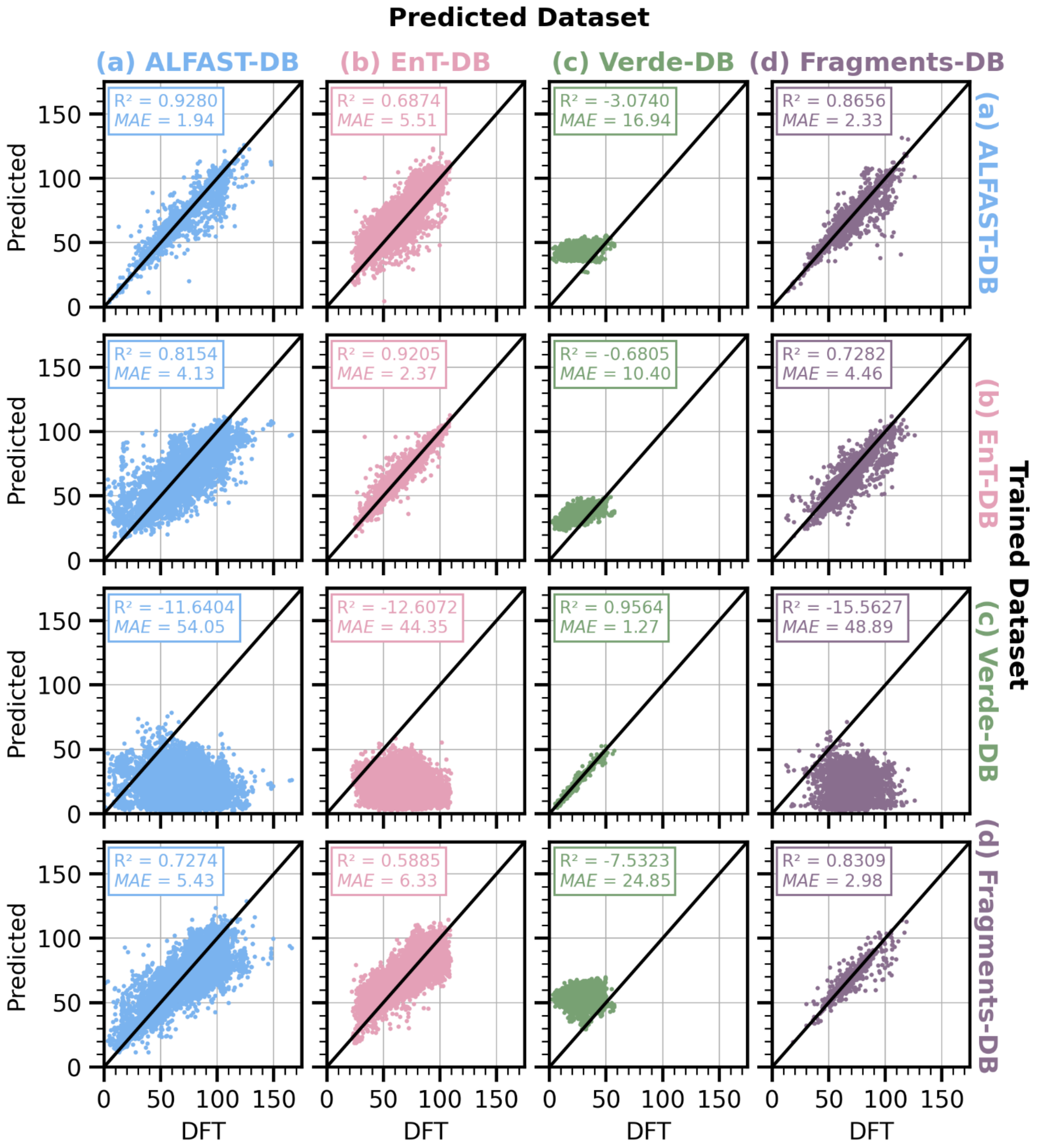

Figure S5: Model extrapolation across datasets (Extended version of Figure 5). Parity plots of adiabatic S_0-T_1 predictions in kcal/mol of each dataset (column) of the same model architecture trained each dataset (row). For example, parity plot "ab" corresponds to the predictions of the "final" ALFAST-DB (trained with ALFAST-DB's training and validation splits as training dataset) and shows the prediction of the full EnT-DB. Parity plots in the diagonal (aa, bb, cc and dd) only show the predictions of each database's test set. The metrics, R^2 and MAE values, correspond to the data presented in each plot, test set metrics for the diagonal elements and extrapolation metrics for the off-diagonal metrics.

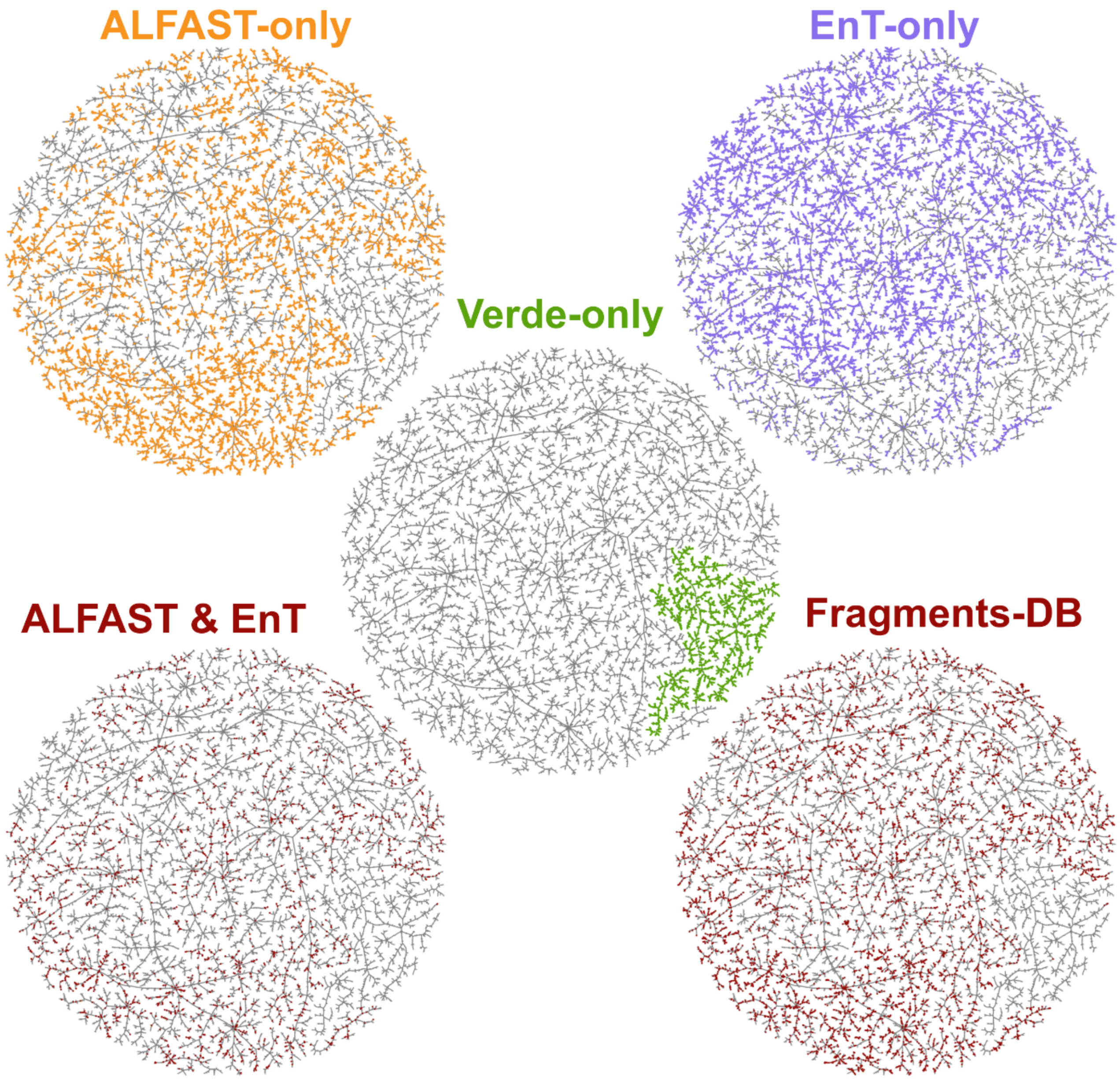

Figure S6: Tree maps of the fragments present in ALFAST-DB, EnT-DB and Verde-DB. Grey lines represent the connections to the most chemically similar fragment based on MHFP encoding and the layout was generated using the LSH forest indexing of the encoded fragments and kept constant across the five tree maps presented.

The tree maps presented in Figure S3 were computed using the python libraries: faerun 0.4.7, mhfp 1.9.6 and the tmap 1.0.6. In these tree maps each fragment is presented as a colored dot and the most similar fragments (based on the MinHash fingerprints of up to 6 bonds representations with a “n_permutations” parameter of 1024) [ref4] are connected through grey edges. The graph layout was generated from the LSHForest and kept constant for the 4 images shown in Figure S3. The nodes representing each fragment were then colored or removed from the graph to generate the four figures, where only the fragments present in each database subgroup are colored. For example, the fragments present in ALFAST-DB but not in EnT-DB nor in Fragments-DB nor in Verde-DB are shown in orange in the top left tree map. In Figure S6 “ALFAST & EnT” stands for fragments which are present in both, ALFAST-DB and EnT-DB but not in Verde-DB. The difference between the “ALFAST & EnT” and the “Fragments-DB” tree map correspond to the increase in chemical coverage of the Fragments-DB.

S6. Analysis of the effect of the theory-shift

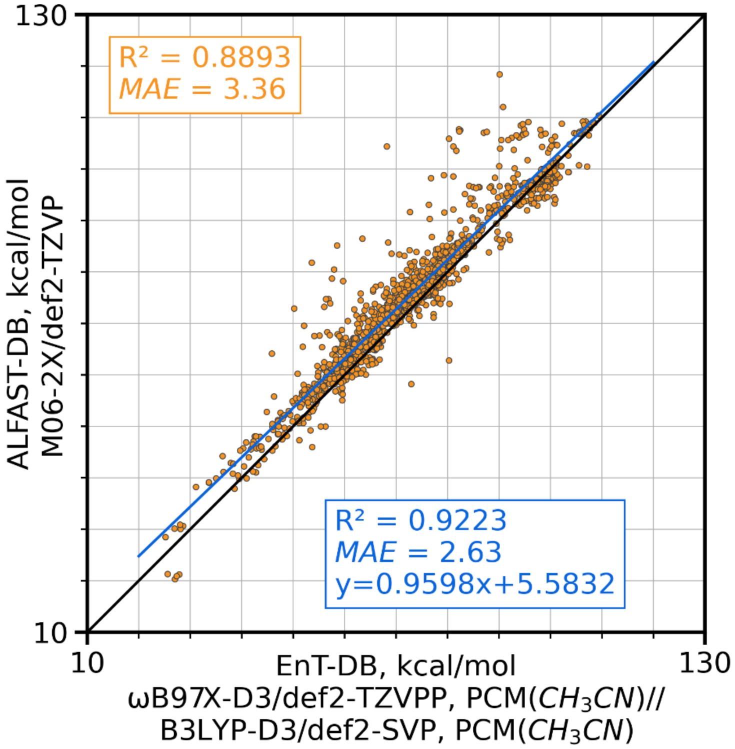

Figure S7: Parity plot and linear fitting of the molecules present in both, the EnT-DB and the ALFAST-DB.

From the Linear fitting obtained from the molecules present in both, EnT-DB and ALFAST-DB we attempted to apply a lineal correction to the parity plots ab and ba presented in figures 5 and S5 (in the manuscript and the present SI respectively. Figure S8A correspond shows the results of applying the lineal correction to the predicted values shown in Figure 5ab (and Figure S5ab), showing a small decrease in the MAE (from 4.13 to 3.95 kcal/mol). Figure S8B shows the results of applying the inverse of the lineal correction shown in Figure S7 to the predicted values in Figure 5ba (and Figure S5ba), showing a larger improvement of the MAE and the R2 than Figure S8A. However, in both cases it is still far from the in-sample performance shown in Figures 5aa and 5bb (and also S5aa and S5bb). The difference in the improvement (0.18 vs 1.2 kcal/mol for Figure S8A and B respectively) can be understood when coupled with the analysis of Figure 6 in the main text. The error distribution of molecules with shared fragments (Figure 6 “shared”, left), which could be expected to be more “in-sample” for the lineal correction, of the ALFAST-DB-trained model has a larger difference with the error distribution of the dataset it was trained on (Figure 6 “all”, left), and therefore it has more room for improvement. On the other hand the EnT-DB-trained model has a smaller difference between the EnT-DB error distribution (Figure 6 “all”, right) and the molecules sharing a fragment across databases (Figure 6 “shared”, right).

Finally, it is to note that a simple lineal correction to shift the theory level is not conceptually correct here. While ALFAST-DB contains the adiabatic S_0-T_1 gap in vacuum, the EnT-DB was designed for the same property in a polar solvent (Acetonitrile). Molecules with a larger change in dipole moment between the two states are expected to display significant differences due to the stabilization of the solvent. This could lead to not only different energies but also guide the optimization to a different exciton localization.

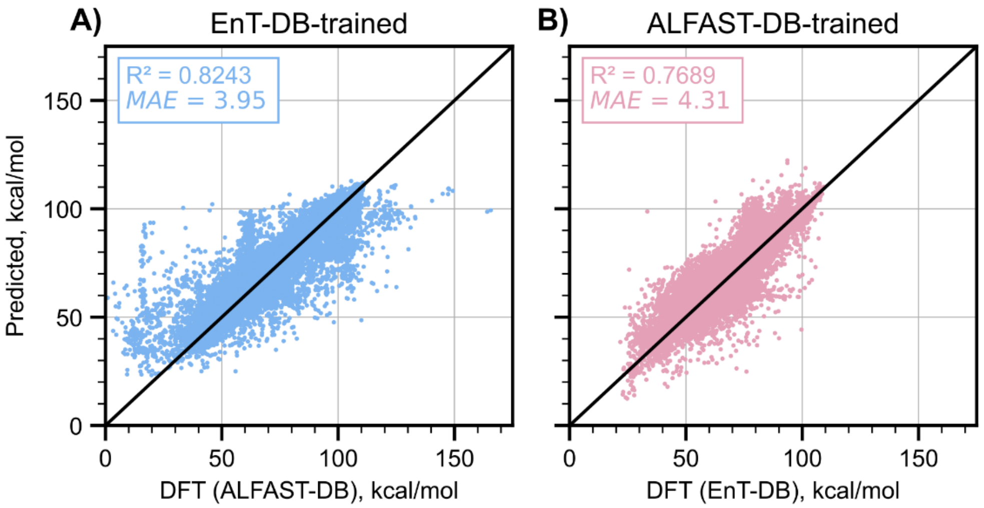

Figure S8: Parity plots of the linearly corrected (using the linear fitting of Figure S7) S0-T1 adiabatic gaps. A) Prediction of ALFAST-DB molecules from a model trained on EnT-DB and applying the lineal correction. B) Prediction of the EnT-DB molecules with a model trained on ALFAST-DB with a consequent lineal correction.

S7. LLM Model Comparison

To further assess the efficacy of our model, we compare its performance to a fine-tuned LLM model. We use a publicly available 108 million parameter RoBERTa model pre-trained on the 480 million SMILES strings available in the ZINC database; the trained model is available at https://huggingface.co/entropy/roberta_zinc_480m. To fine tune the model, we use an ANN readout regression head consisting of 4 linear layers each separated by a ReLU activation function (referred to henceforth as the "readout model"); the final output layer consists of a single linear layer. An embedding dimension of 128 was used for all linear layers. All model layers were implemented in pytorch 2.5.1. The huggingface transformers library was used for loading the pretrained model and generating embeddings for smiles strings corresponding to molecules in the ALFAST database; the generated embeddings were used as inputs for the readout regression model. We follow the train-validation-test split strategy used to train the final Δ-learning predictor model to train and evaluate the readout model. The readout model was trained for 200 epochs using an AdamW optimizer with a learning rate of 10^{-3} and an MAE loss function as implemented in pytorch. The trained readout model achieves an MAE of 6.17 kcal/mol on the held out test set. Prediction results are shown along with prediction results from the Δ-learning model in Figure S9:

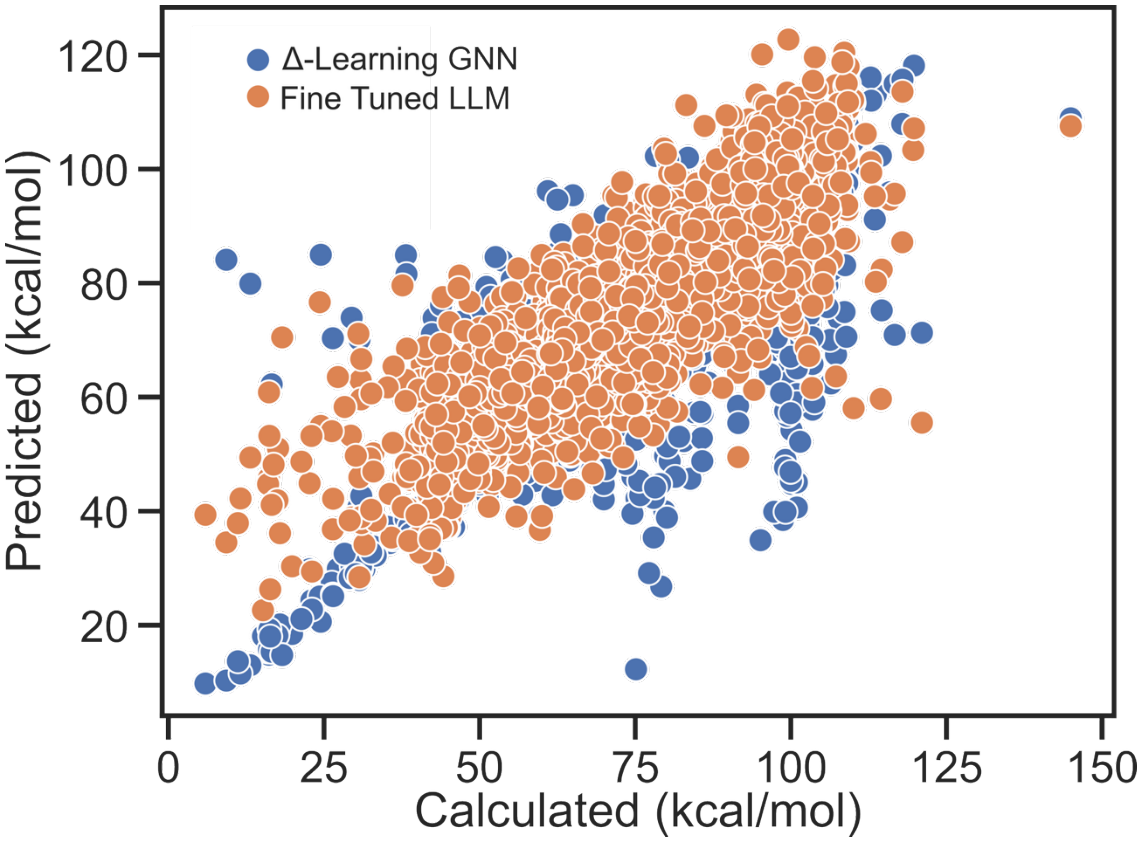

Figure S9: Prediction results for the fine-tuned LLM model vs. Δ-learning GNN model predictions

S8. XYZ coordinates

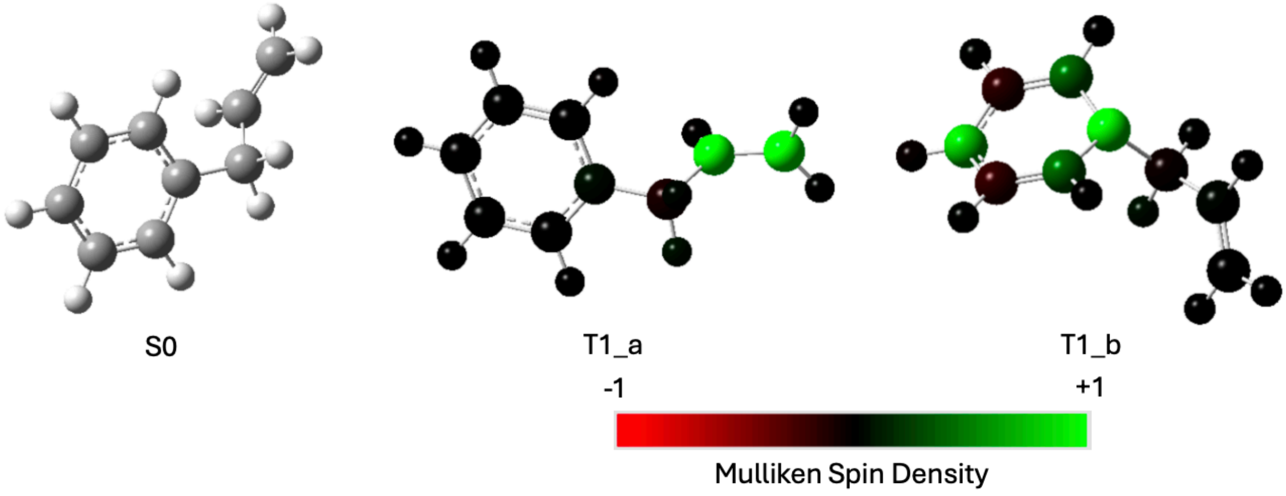

Mol1-S0

C	2.63141700	0.51890500	0.14912300
C	2.34470700	-0.83737600	0.18097800
C	1.03914500	-1.27734400	0.00351000
C	0.00486600	-0.37247300	-0.20829300
C	0.30320400	0.98822000	-0.23337100
C	1.60498800	1.43176800	-0.05901800
H	3.64752400	0.86443200	0.28806500
H	3.13725300	-1.55615300	0.34549500
H	0.82037500	-2.33861700	0.02923700
H	-0.49856200	1.70164700	-0.38588800

H	1.82025500	2.49242600	-0.08323200
C	-1.41755500	-0.84476000	-0.41827900
H	-1.45483000	-1.92561200	-0.25965900
H	-1.71753800	-0.66289200	-1.45386400
C	-2.39181000	-0.16883200	0.50129500
H	-2.20175600	-0.29088100	1.56420000
C	-3.42322200	0.55886000	0.10089800
H	-3.63255800	0.70508400	-0.95303000
H	-4.09459700	1.02875800	0.80761800

Mol1-T1_a

C	-2.72915200	0.32473300	-0.20135400
C	-2.28720500	-0.98924600	-0.20637100
C	-0.94647000	-1.27451300	0.02129800
C	-0.03165800	-0.25503300	0.25729700
C	-0.48655700	1.06247900	0.25729100
C	-1.82290700	1.35214900	0.03163900
H	-3.77277000	0.54935800	-0.37897900
H	-2.98525000	-1.79610200	-0.38955800
H	-0.60602400	-2.30344800	0.01447800
H	0.22100500	1.86493700	0.43495400
H	-2.15995000	2.38087100	0.03748700
C	1.42667200	-0.54974000	0.50442500
H	1.58883900	-1.63563600	0.46613100
H	1.69787600	-0.25236200	1.52427000
C	2.34314400	0.14172600	-0.45981500
H	1.94530900	0.38739900	-1.44022800
C	3.76973400	0.23240600	-0.21127600
H	4.44337000	-0.58182000	-0.45735700
H	4.21398900	1.11704200	0.23000000

Mol1-T1_b

C	-2.35517600	0.65457700	0.47855900
C	-2.30360100	-0.76248900	0.25677000
C	-1.21841400	-1.32129400	-0.31523500
C	-0.00542200	-0.48723700	-0.49377800
C	-0.25611200	0.96557000	-0.67348700
C	-1.35112100	1.50050200	-0.09306400
H	-3.22880500	1.09328900	0.93827700
H	-3.17700500	-1.36149900	0.48585300
H	-1.19472100	-2.37232000	-0.57907900
H	0.46753600	1.58010700	-1.19429900
H	-1.52065400	2.57044100	-0.11982000
C	1.18296900	-0.84872300	0.36537300
H	1.23396400	-1.93832300	0.45105300
H	1.03406000	-0.45335100	1.38064700
C	2.47389900	-0.32162900	-0.19168600
H	2.75886500	-0.70666200	-1.16712800
C	3.24609700	0.56932600	0.41063800
H	2.98196100	0.97786700	1.37962900
H	4.16607800	0.91883500	-0.03966800

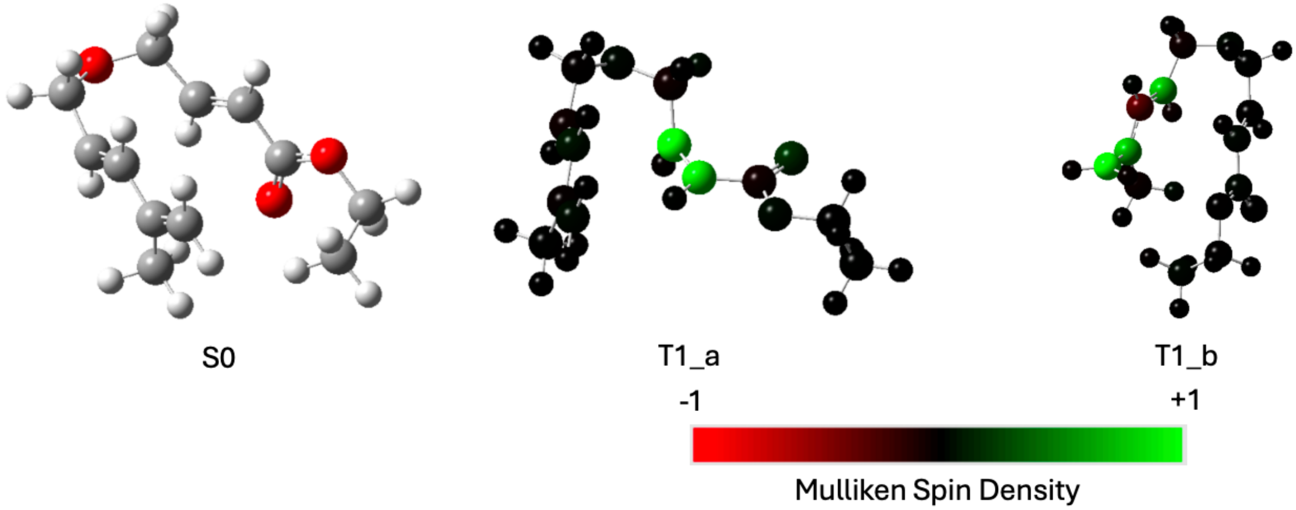

Mol2-S0

O	-3.46629700	-1.06066400	0.25628900
C	-3.50518000	0.29535400	-0.16264900
H	-3.59550400	0.34686600	-1.25496200
H	-4.41866700	0.69798900	0.27714400
C	-2.45795700	-1.83529300	-0.35222400
H	-2.73814000	-2.87485600	-0.16347700
H	-2.45041000	-1.68134800	-1.43881300
C	-2.30012400	1.06076800	0.28858900
H	-2.13935300	1.10213300	1.36158400
C	-1.40408700	1.57562100	-0.54919000
H	-1.58214600	1.49431400	-1.61938500
C	-1.09138700	-1.59086900	0.21686800
H	-1.03977100	-1.41337100	1.28762300
C	0.03642000	-1.59046800	-0.48088800
H	0.06330000	-1.74259500	-1.55276200
C	-0.12662300	2.17547900	-0.16018800
C	0.72417000	2.57776900	-1.10656500
H	0.47537300	2.49375500	-2.15753600
H	1.68778800	3.00329700	-0.85666500
C	0.20657700	2.25982900	1.30038600
H	0.26761800	1.25788000	1.73428200
H	1.16344600	2.75702300	1.45047100
H	-0.56029700	2.81444900	1.84519400
C	1.31873900	-1.29137100	0.19169800
O	1.44570300	-1.02603300	1.36047300
O	2.34145900	-1.33302600	-0.67427000
C	3.62633200	-0.97018200	-0.15293000
H	4.33996900	-1.40455500	-0.85035200
H	3.75307400	-1.42885000	0.82703500
C	3.77011300	0.53503100	-0.07648000
H	4.76637900	0.79566500	0.28245200
H	3.62595500	0.98046100	-1.06085100
H	3.03251300	0.94952200	0.61052000

Mol2-T1_a

O	-2.04072400	-2.67881100	0.06453800
C	-2.93031200	-1.73684100	-0.50377800
H	-2.78256800	-1.68850800	-1.59036900
H	-3.92817900	-2.13559300	-0.31185500
C	-0.67726900	-2.37029500	-0.15543800

H	-0.13389500	-3.30622900	0.00134900
H	-0.51818400	-2.06161000	-1.19642500
C	-2.79156500	-0.36706200	0.08650100
H	-2.91425100	-0.30382900	1.16346600
C	-2.52221100	0.72356600	-0.63058300
H	-2.39872600	0.62676100	-1.70686900
C	-0.15325400	-1.31944900	0.77470700
H	-0.24653700	-1.50571100	1.83930100
C	0.64421400	-0.20339600	0.30501400
H	0.20956700	0.77642500	0.12968100
C	-2.33378000	2.06878500	-0.08176100
C	-2.04017200	3.08441900	-0.89548500
H	-1.95364500	2.93806900	-1.96516700
H	-1.88028500	4.08570300	-0.51681400
C	-2.44856000	2.24811500	1.40423300
H	-1.72767400	1.61244200	1.92575500
H	-2.26582400	3.28285000	1.68679500
H	-3.44217400	1.96150000	1.75513500
C	2.05759200	-0.38363100	0.00314500
O	2.64219000	-1.44041900	0.08829900
O	2.63867900	0.76220800	-0.39849300
C	4.03443700	0.68665900	-0.71144200
H	4.21947700	1.54811500	-1.35050400
H	4.22145400	-0.22741000	-1.27460200
C	4.88197900	0.73667100	0.54225300
H	5.93892600	0.74193500	0.27372000
H	4.66570000	1.64038200	1.11196800
H	4.68905800	-0.13436400	1.16649300

Mol2-T1_b

O	-3.50939500	-0.92463900	0.30168500
C	-3.50361600	0.48430600	0.09029400
H	-3.72096900	0.69806300	-0.96374000
H	-4.33389900	0.85398000	0.69430600
C	-2.57534800	-1.63294500	-0.48280800
H	-2.93443700	-2.66515000	-0.50452700
H	-2.56722500	-1.25650300	-1.51365600
C	-2.21176700	1.11661700	0.48467200
H	-1.93157500	1.02695900	1.52836000
C	-1.33229000	1.65194500	-0.42436600
H	-1.64768200	1.70203100	-1.46426300
C	-1.18641800	-1.61469000	0.08482100
H	-1.09095300	-1.81773100	1.14791400
C	-0.09116400	-1.37854200	-0.62606900
H	-0.12875900	-1.13318100	-1.68061000
C	-0.02524200	2.03463100	-0.14264000
C	0.87333200	2.45178900	-1.22141600
H	1.03612200	3.49947500	-1.44322900
H	1.44574800	1.71880200	-1.77694400
C	0.51120900	1.96234200	1.25920200
H	0.61475700	0.91931400	1.58307000
H	1.48919200	2.43514200	1.33007100
H	-0.16328500	2.45289800	1.96524900
C	1.23672500	-1.34346300	0.02096600
O	1.46450600	-1.61687500	1.17148300
O	2.18126600	-0.93885900	-0.84515900

C	3.51850300	-0.85008200	-0.33466500
H	4.15000500	-0.86925300	-1.22085300
H	3.72171700	-1.73197900	0.27247700
C	3.72405800	0.41931900	0.46413400
H	4.77215500	0.50721800	0.75274900
H	3.45389800	1.29349400	-0.13031900
H	3.11627900	0.40204100	1.36712200

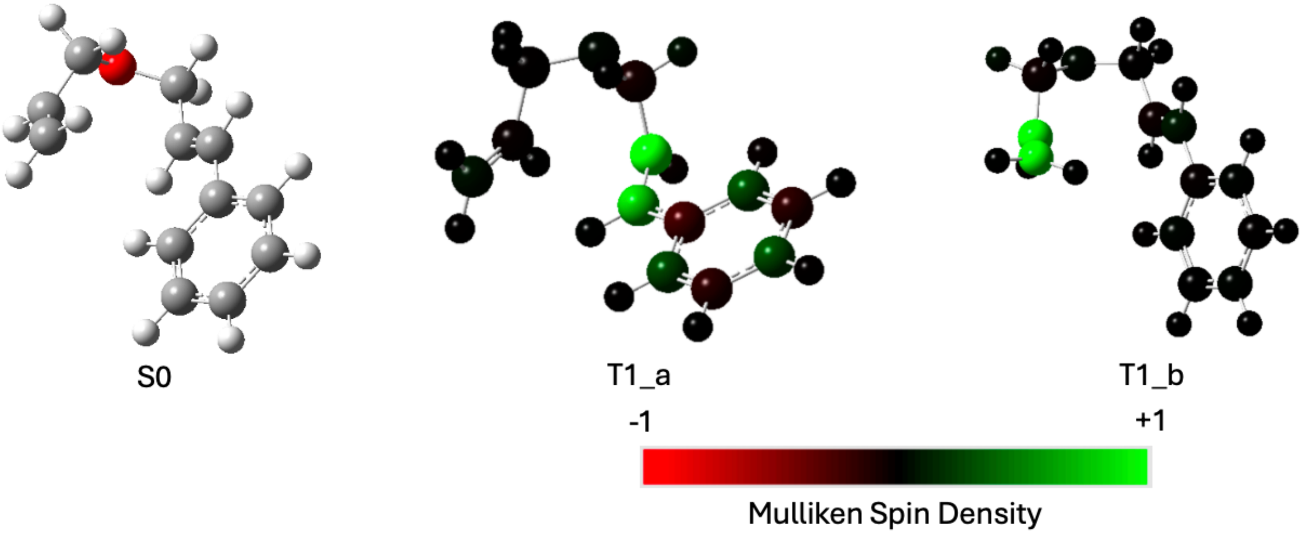

Mol3-S0

O	3.40146600	-0.61371800	-0.39546100
C	2.36803100	-1.57937700	-0.46241300
H	2.63574400	-2.22119300	-1.30396200
H	2.36494800	-2.19188900	0.44824700
C	3.23817600	0.34517700	0.63531200
H	2.66295500	-0.07199500	1.46975600
H	4.24419100	0.57096400	0.99810700
C	1.01472500	-0.97373700	-0.66632600
H	0.91467000	-0.32213600	-1.52886500
C	-0.01160800	-1.17387800	0.15427000
H	0.12182000	-1.84996100	0.99610400
C	2.59110000	1.60620100	0.14090900
H	3.08269000	2.07655000	-0.70579800
C	1.51015100	2.15250900	0.67543000
H	0.99266000	1.68068300	1.50392200
H	1.09781900	3.08205500	0.30411200
C	-1.33967200	-0.55524500	0.04035600
C	-1.54783400	0.61762300	-0.68986200
C	-2.42768100	-1.13082500	0.69716400
C	-2.81084800	1.17906400	-0.77862300
H	-0.70799800	1.10231000	-1.17211600
C	-3.69311000	-0.57119200	0.60667200
H	-2.27611500	-2.03276900	1.27864300
C	-3.88967000	0.58489400	-0.13493200
H	-2.95452900	2.08995900	-1.34556800
H	-4.52601600	-1.03671500	1.11763600
H	-4.87512600	1.02659800	-0.20428200

Mol3-T1_a

O	-2.95462200	-1.47912100	-0.13924200
C	-1.55666100	-1.37756700	-0.34093100
H	-1.19986900	-2.41298200	-0.35943000
H	-1.34325500	-0.93468400	-1.32213200
C	-3.67032500	-0.32059700	-0.51144200
H	-3.46003000	-0.06439900	-1.55815700
H	-4.72340300	-0.59990600	-0.43596800
C	-0.85379400	-0.60298500	0.72996700
H	-1.07694300	-0.87801600	1.75639700
C	0.09846000	0.46014500	0.44297000
H	-0.27623000	1.48046900	0.39443100
C	-3.40300700	0.87059400	0.36131100
H	-3.51632200	0.70885600	1.42941500
C	-3.08663200	2.06930500	-0.10604200
H	-2.96502500	2.24181000	-1.17003800
H	-2.94302600	2.91789100	0.55040100
C	1.48162400	0.25803500	0.20945200
C	2.06752300	-1.02783200	0.24791700
C	2.32795100	1.35460400	-0.07486600
C	3.41699000	-1.19906600	0.01371900
H	1.44149400	-1.88382800	0.46947900
C	3.67451500	1.17287400	-0.30756900
H	1.89830700	2.34880100	-0.10759500
C	4.23097100	-0.10445400	-0.26603700
H	3.84495400	-2.19288000	0.04880300
H	4.30269600	2.02773400	-0.52331800
H	5.28795000	-0.24422800	-0.44904900

Mol3-T1_b

O	3.44287700	-0.58504300	-0.49409700
C	2.41762500	-1.54529000	-0.33212800
H	2.65236400	-2.33492600	-1.04856400
H	2.45872800	-1.97393600	0.67773500
C	3.34932900	0.49995300	0.41292800
H	3.13124500	0.13329900	1.42444400
H	4.35118200	0.94207000	0.42667600
C	1.04785000	-0.99707500	-0.59113700
H	0.90214200	-0.53326700	-1.56206300
C	0.04662200	-1.06717200	0.28263200
H	0.22033700	-1.56791200	1.23248700
C	2.32836000	1.52059700	0.01329000
H	2.38646100	1.88365200	-1.00917600
C	1.37320900	2.08721900	0.94505000
H	0.37460200	1.67455200	1.04556600
H	1.61886500	2.94381200	1.56279300
C	-1.30352900	-0.52028600	0.08819800
C	-1.56359500	0.49819400	-0.83273100
C	-2.36220000	-1.00569100	0.85672400
C	-2.84651300	0.99338700	-0.99575800
H	-0.74794900	0.92085600	-1.40718000
C	-3.64793000	-0.51304100	0.69208700
H	-2.17118000	-1.78521400	1.58491700
C	-3.89542900	0.48636200	-0.23789400
H	-3.02893400	1.78600200	-1.70997500
H	-4.45693500	-0.90787000	1.29302800

H	-4.89673800	0.87627300	-0.36547500

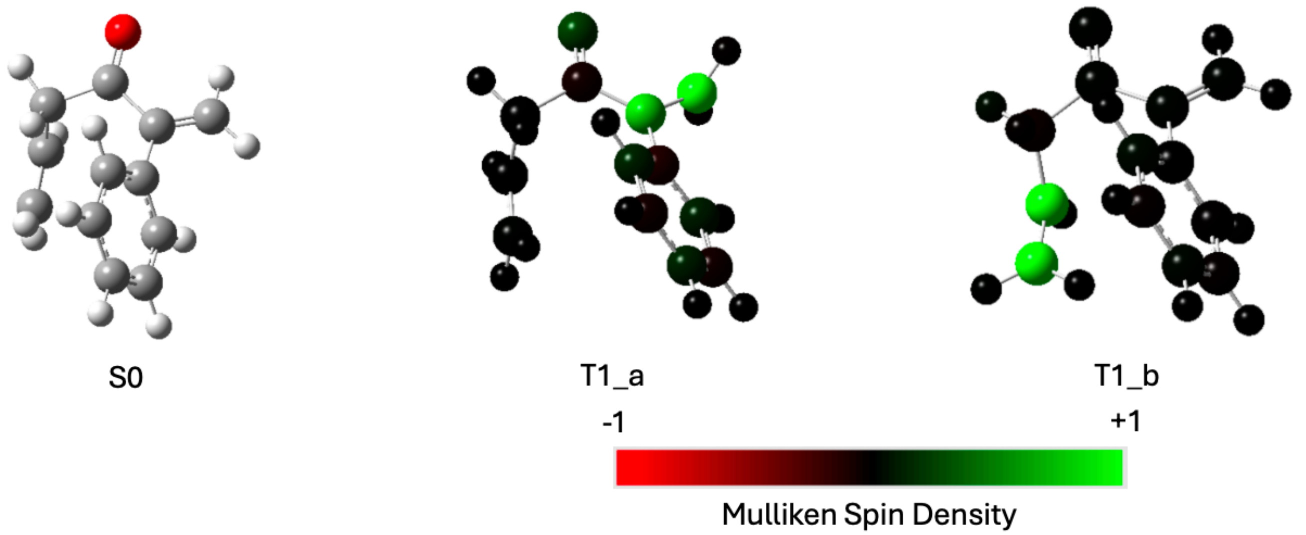

Mol4-S0

C	1.16324400	-2.04256600	-1.14670300
H	2.19941800	-2.32207600	-1.28619000
C	0.84156100	-1.07544400	-0.29207300
C	1.97407400	-0.38322800	0.43115900
O	3.02092200	-0.94602700	0.62794700
H	0.40399500	-2.57366100	-1.70714500
C	-0.55937600	-0.64636100	-0.07008700
C	-1.40153900	-0.37981400	-1.14830300
C	-1.06629100	-0.51964200	1.22257100
C	-2.71698000	0.00600300	-0.94000500
H	-1.00644900	-0.45170800	-2.15407700
C	-2.38170900	-0.13354000	1.43328100
H	-0.42787400	-0.74134400	2.07038100
C	-3.21015400	0.13351500	0.35159500
H	-3.35611100	0.21662900	-1.78775100
H	-2.76159700	-0.04633600	2.44293400
H	-4.23530200	0.43911500	0.51484500
C	1.78120600	1.07809200	0.81260300
H	0.90703600	1.18833100	1.45456100
H	2.67204900	1.37667100	1.36462500
C	1.60072400	1.91721400	-0.42207400
H	2.46290600	1.98669000	-1.07876500
C	0.47371000	2.53171500	-0.74654000
H	-0.40686300	2.46343300	-0.11630800
H	0.39059400	3.11681100	-1.65322400

Mol4-T1_a

C	1.03232600	-2.38851800	-0.64035900
H	1.37780100	-2.43507500	-1.66500900
C	0.77484800	-1.10020000	0.00115200
C	1.95219100	-0.35376800	0.46233600
O	3.02630200	-0.92172400	0.56574000
H	1.13806200	-3.27956300	-0.03601300
C	-0.59655000	-0.61403200	0.09991000
C	-1.49761700	-0.86306800	-0.94531500
C	-1.06986800	0.06349500	1.23171400

C	-2.79969800	-0.39931700	-0.88654200
H	-1.15519300	-1.40807800	-1.81585600
C	-2.37898100	0.51011700	1.29608900
H	-0.41763000	0.20093400	2.08394400
C	-3.24530400	0.29459000	0.23194500
H	-3.47262900	-0.58318900	-1.71393700
H	-2.72792500	1.01943700	2.18500700
H	-4.26590300	0.65065000	0.28095200
C	1.87459400	1.16125500	0.63635100
H	1.04476900	1.46706100	1.26778100
H	2.81018400	1.45999900	1.10873100
C	1.73189200	1.79982800	-0.71782700
H	2.58624600	1.69686000	-1.38056100
C	0.64201600	2.42442700	-1.13769500
H	-0.23098800	2.52413600	-0.50130700
H	0.58369700	2.85177600	-2.13021100

Mol4-T1_b

C	1.44204600	-2.24134600	-0.57999600
H	2.49897500	-2.45178600	-0.68048800
C	1.04008700	-1.07617500	-0.08021100
C	2.10694900	-0.07683400	0.30883000
O	3.24488100	-0.42598200	0.49259500
H	0.73003000	-2.99853300	-0.88356100
C	-0.39471100	-0.72939100	0.05912100
C	-1.26004600	-0.85369700	-1.02684200
C	-0.90849600	-0.28539900	1.27685100
C	-2.60487100	-0.53784400	-0.90057700
H	-0.86310400	-1.17965200	-1.98047400
C	-2.25272000	0.03159100	1.40484900
H	-0.25318000	-0.20518100	2.13618900
C	-3.10461500	-0.09051200	0.31539800
H	-3.26196000	-0.63301700	-1.75540700
H	-2.63638700	0.37062600	2.35831900
H	-4.15247900	0.16095900	0.41360600
C	1.71366600	1.39557500	0.39463500
H	1.10341900	1.54830400	1.28744400
H	2.65013900	1.94301600	0.53144200
C	0.96007300	1.86523700	-0.81121400
H	1.44983900	1.75546800	-1.77458700
C	-0.33094400	2.52183500	-0.72513100
H	-0.40593200	3.59660600	-0.60095700
H	-1.25692700	1.96280700	-0.80657000

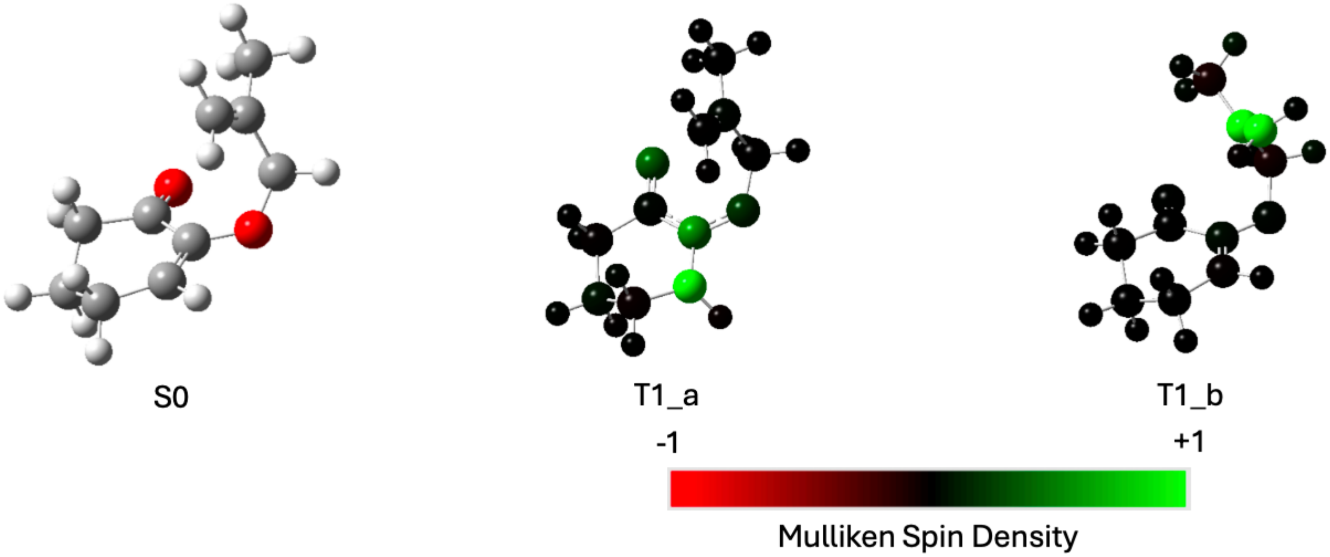

Mol5-S0

C	-1.53832700	-1.37067000	-0.07771100
C	-0.51745300	-0.60527400	-0.47825500
C	-1.75151500	1.40080700	0.46685900
C	-3.03169700	0.62648100	0.17545900
C	-2.82813300	-0.85223200	0.47835400
H	-1.41134100	-2.44486800	-0.16350500
H	-1.47331600	1.27554600	1.51987100
H	-1.84815100	2.46662600	0.26714100
H	-3.86087200	1.02808900	0.75876800
H	-3.28919500	0.74637000	-0.88027300
H	-2.83208500	-1.01178800	1.56397700
H	-3.65886000	-1.44299300	0.08667200
C	-0.60032000	0.88162200	-0.36513500
O	0.19375500	1.62545800	-0.89134900
O	0.56301500	-1.21250700	-1.03838000
C	1.84967100	-0.60940900	-0.95815400
H	1.90226800	0.25757100	-1.61471100
H	2.52692000	-1.37363900	-1.34672400
C	3.33408900	0.84911800	0.46040600
H	2.92621000	1.77600600	0.04957000
H	3.68790300	1.03303300	1.47324600
H	4.19015600	0.57091200	-0.16049400
C	2.28594600	-0.22113900	0.43010900
C	1.78605500	-0.78785300	1.52076900
H	2.12719900	-0.49433500	2.50572700
H	1.02910500	-1.55883700	1.46235700

Mol5-T1_a

C	-1.70678200	-1.40464100	-0.24751200
C	-0.54916000	-0.52510900	-0.52408900
C	-1.91971300	1.46631500	0.19424600
C	-3.12066400	0.52593500	0.20123000
C	-2.71022100	-0.87158600	0.70651100
H	-2.01453700	-2.07876700	-1.03934200
H	-1.69333000	1.78816900	1.21560800
H	-2.12934600	2.37498300	-0.37150200
H	-3.91351400	0.93698900	0.82848400
H	-3.52866500	0.42734000	-0.80805100

H	-2.26794900	-0.76200900	1.70391700
H	-3.57177000	-1.53257900	0.78842400
C	-0.61677200	0.89301800	-0.35277700
O	0.33994000	1.65661700	-0.54990200
O	0.51829400	-1.21412000	-0.90093500
C	1.81699700	-0.61608300	-0.96715600
H	1.79222600	0.25605100	-1.61600900
H	2.44080100	-1.38915100	-1.41960400
C	3.46105600	0.76806100	0.33271900
H	3.06322200	1.71005000	-0.05207600
H	3.89183100	0.93781400	1.31761700
H	4.25788800	0.44513300	-0.34295900
C	2.35892400	-0.24336500	0.38704300
C	1.89031800	-0.77150900	1.50802000
H	2.30220000	-0.48833700	2.46803300
H	1.08117200	-1.49187400	1.50474800

Mol5-T1_b

C	-1.28438600	-1.39267600	-0.27762000
C	-0.43589300	-0.43389200	-0.66112000
C	-1.81470200	1.23442800	0.67067400
C	-2.99593900	0.31337200	0.38745700
C	-2.55978400	-1.14458700	0.46773700
H	-1.02406000	-2.41427500	-0.53443900
H	-1.42020200	1.03367500	1.67402200
H	-2.07986400	2.28927000	0.62050900
H	-3.80895100	0.50985800	1.08709700
H	-3.37542400	0.51902900	-0.61698400
H	-2.41699500	-1.43263800	1.51683600
H	-3.34233100	-1.80134400	0.08204200
C	-0.68695500	0.99444400	-0.30917100
O	-0.02431300	1.89786800	-0.76252900
O	0.63696600	-0.75724100	-1.43046300
C	1.90222900	-0.17647800	-1.07584700
H	1.96657400	0.84021700	-1.46070000
H	2.62537700	-0.80505400	-1.60630200
C	2.81368700	0.97902800	1.03571900
H	2.44280600	1.90939300	0.60077600
H	2.63716100	0.99400700	2.11221200
H	3.90263800	0.95789400	0.89264700
C	2.15178400	-0.19918400	0.39854300
C	2.06466400	-1.47215600	1.10300900
H	1.16528400	-1.77148700	1.62740600
H	2.89852100	-2.16735800	1.11251400

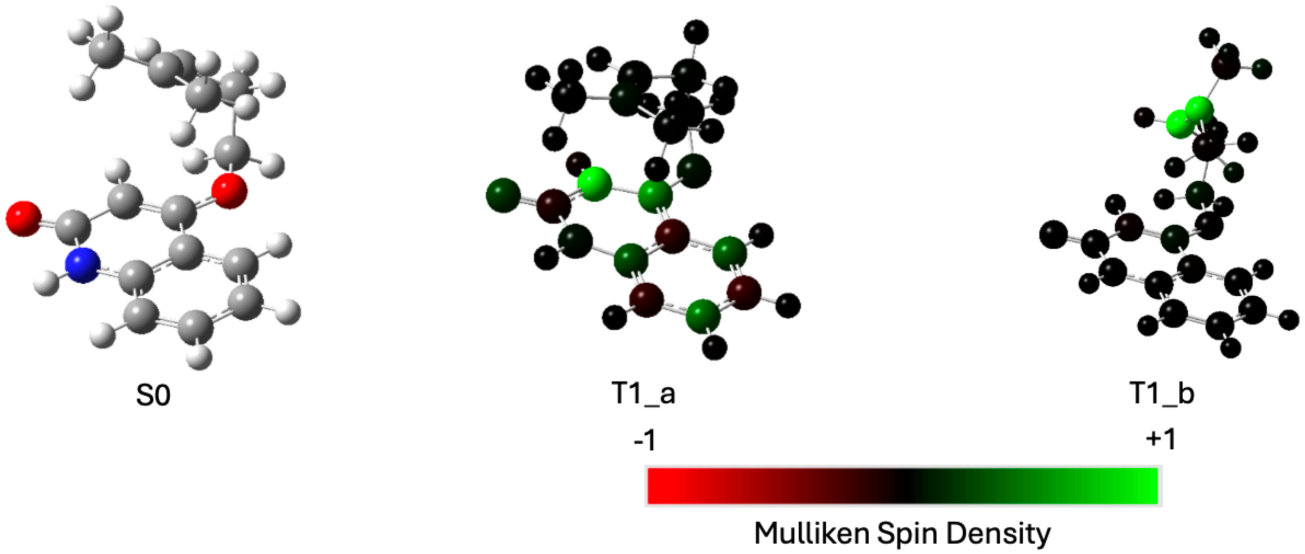

Mol6-S0

C	0.37265400	1.10048600	-1.03969500
C	-0.02377900	-0.18383500	-0.88993500
C	-1.33096100	-0.49836000	-0.33596900
C	-2.16253000	0.56870700	0.03054200
C	-0.48277800	2.21177600	-0.66475300
H	-1.12513000	-2.62301800	-0.43211600
H	1.34751000	1.38121000	-1.40267100
C	-1.77767800	-1.80976100	-0.14750200
C	-3.42498300	0.31206000	0.57487200
H	-2.30954800	2.63060400	0.11398300
C	-3.84503000	-0.98855500	0.75128400
C	-3.02216000	-2.05787600	0.39151500
H	-4.06164000	1.14256000	0.85396700
H	-4.82311600	-1.17814200	1.17396400
H	-3.36021600	-3.07486600	0.53435400
O	-0.17673300	3.38499900	-0.76860600
O	0.68677800	-1.27696500	-1.20694600
N	-1.71855900	1.85548200	-0.14905100
C	1.97451100	-1.19060500	-1.81245600
H	2.00436600	-2.01692600	-2.52299900
H	2.07351000	-0.25985900	-2.37194700
C	3.07897300	-1.35345800	-0.77362300
H	2.90328500	-2.28960100	-0.24174900
H	4.02028100	-1.47148000	-1.31610600
C	3.17728400	-0.19019300	0.16989600
H	3.80596100	0.63149100	-0.16372400
C	2.52306600	-0.03740900	1.31896400
C	1.57189800	-1.04267100	1.89877300
H	1.52788000	-1.97239600	1.33678200
H	1.83926600	-1.26719600	2.93426700
H	0.56170000	-0.62024200	1.91857500
C	2.63676800	1.23436400	2.10788200
H	1.66462500	1.73515000	2.14841900
H	2.93370500	1.02926600	3.13945900
H	3.35557700	1.92278500	1.66653900

Mol6-T1_a

C	0.83952200	0.45272800	-1.54872800
C	0.08882000	-0.72096300	-1.04553600
C	-1.17069300	-0.56085000	-0.50405000
C	-1.65902200	0.76981200	-0.27160100
C	0.37538000	1.78330300	-1.24364800
H	-1.63990100	-2.65256900	-0.26303200
H	1.64496300	0.36200500	-2.25978900
C	-2.00279400	-1.64731000	-0.10168200
C	-2.89663500	0.96925500	0.29460800
H	-1.15837100	2.78736900	-0.38213000
C	-3.69698500	-0.11776800	0.66526600
C	-3.23327000	-1.41986700	0.46875400
H	-3.24076400	1.98194900	0.46921100
H	-4.66677100	0.05807700	1.10891000
H	-3.84925600	-2.25921300	0.76385000
O	0.98243200	2.79823500	-1.54779000
O	0.59063600	-1.96086100	-1.22879800
N	-0.82328700	1.85213200	-0.55834600
C	1.97951100	-2.16212100	-0.98505400
H	2.14263000	-3.21524200	-1.20479000
H	2.58079700	-1.56927800	-1.68087300
C	2.36510300	-1.84356200	0.45975100
H	1.65201400	-2.33612800	1.12022400
H	3.34006200	-2.30830100	0.63657900
C	2.47383900	-0.37427000	0.73107000
H	3.28387300	0.12536100	0.20368400
C	1.71136700	0.37930400	1.53126900
C	0.54610700	-0.12528000	2.32722400
H	0.31904600	-1.17415800	2.15395500
H	0.73087700	0.02204200	3.39488900
H	-0.35002500	0.45131300	2.07868400
C	1.98788400	1.84686100	1.68542700
H	1.10087700	2.43160000	1.42849000
H	2.23202500	2.07948900	2.72564300
H	2.80757600	2.17614600	1.04919800

Mol6-T1_b

C	0.03649600	1.64688800	0.54924100
C	0.19033400	0.35474500	0.91850900
C	1.34960900	-0.40828900	0.48704400
C	2.29295400	0.23433500	-0.32569200
C	1.01534700	2.32468500	-0.28259900
H	0.81089600	-2.23102400	1.46381100
H	-0.81693200	2.24674300	0.81777800
C	1.54765500	-1.74694800	0.83835700
C	3.41822000	-0.46553400	-0.77264800
H	2.76945500	2.01971200	-1.25633200
C	3.59402600	-1.78407800	-0.41299500
C	2.65809000	-2.43311900	0.39496800
H	4.14262000	0.03884600	-1.40013500
H	4.46756900	-2.31826600	-0.76342300
H	2.80430900	-3.46809000	0.67133500
O	0.93160900	3.48412200	-0.64087800
O	-0.65210100	-0.35741300	1.68282400
N	2.09340300	1.54759100	-0.67374800

C	-1.86434200	0.20650900	2.17077400
H	-2.00488500	-0.23609000	3.15698000
H	-1.76619300	1.28464800	2.29665000
C	-3.04322400	-0.15497700	1.26850200
H	-3.10607200	-1.24532900	1.22257000
H	-3.94690000	0.19809600	1.78067200
C	-2.96455300	0.40880400	-0.11761000
H	-3.09636200	1.48070400	-0.23644100
C	-2.83101500	-0.44583000	-1.29609800
C	-4.04419900	-1.16933500	-1.79146600
H	-4.93152200	-0.53555600	-1.74331800
H	-3.91209400	-1.50727600	-2.82105600
H	-4.25780200	-2.06319200	-1.18672000
C	-1.47484400	-0.94618400	-1.67984900
H	-1.06807800	-1.63004000	-0.91971500
H	-1.50213200	-1.48621700	-2.62720000
H	-0.75909600	-0.12451500	-1.76542600

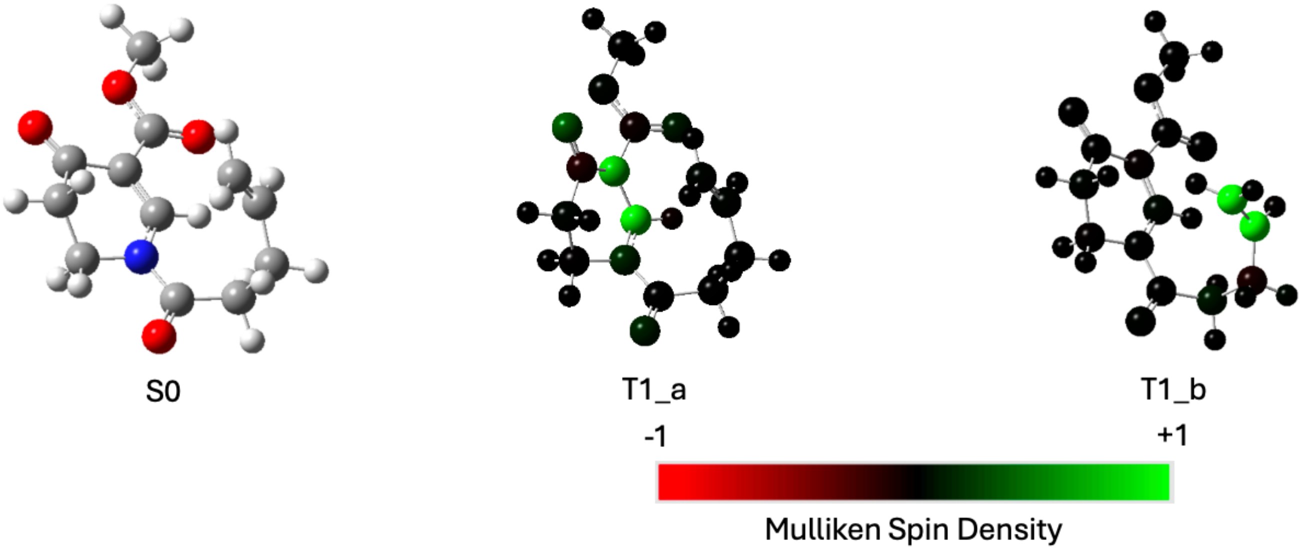

Mol7-S0

C	1.17787900	0.32477600	-0.22036800
C	0.17968300	2.36928600	0.81590200
C	-0.90589400	2.24454200	-0.23608000
C	-0.08813800	-0.00448700	-0.57474800
H	-0.16828100	1.93346800	1.75801900
H	-1.84218700	2.68679800	0.08849600
H	-0.60028500	2.73736500	-1.16382300
H	-0.25541500	-1.00678600	-0.93764200
H	0.42846100	3.41341700	0.99349600
C	1.44736400	1.63111300	0.41648700
C	2.17188300	-0.76013600	-0.41632600
C	-2.47212300	0.43306200	-0.81105400
N	-1.15760400	0.83117300	-0.53697900
O	2.53842100	2.08044400	0.65935100
O	1.88693400	-1.85062100	-0.85465600
O	-3.36088800	1.24827400	-0.77914900
O	3.40303700	-0.42404400	-0.05170000
C	4.38729400	-1.44161100	-0.21549500
H	4.45515500	-1.73834800	-1.26112900
H	5.32076900	-1.00188300	0.12109500
H	4.13387200	-2.31522100	0.38345400

C	-2.77116100	-1.02621500	-1.07822700
H	-1.98464500	-1.53749300	-1.63006600
H	-3.66437400	-1.01994200	-1.69984100
C	-3.08290100	-1.76568100	0.23910300
H	-3.86987500	-1.22131200	0.76457200
H	-3.48292600	-2.74765100	-0.02355100
C	-1.89043300	-1.93212800	1.13135900
H	-1.08987900	-2.56757200	0.75803600
C	-1.75551300	-1.34914300	2.31309300
H	-2.53411500	-0.71046400	2.71619300
H	-0.87071300	-1.49527800	2.91890900

Mol7-T1_a

C	0.98215800	0.42680500	-0.36145400
C	-0.34015000	2.30746400	0.67801500
C	-1.21396600	2.14227000	-0.55740200
C	-0.23511400	0.02996000	-1.13655300
H	-0.88867900	1.97310600	1.56238900
H	-2.19922500	2.57353900	-0.41502800
H	-0.74765400	2.59946300	-1.43119100
H	-0.30428600	-1.00685500	-1.41180900
H	-0.07152200	3.35191500	0.83639300
C	0.94839200	1.50740700	0.60178400
C	2.12773200	-0.49684800	-0.49152300
C	-2.64310600	0.14244800	-0.66367700
N	-1.38191100	0.71283300	-0.83078100
O	1.89293000	1.77566500	1.33142500
O	1.96506500	-1.66907200	-0.74103100
O	-3.60359800	0.83961100	-0.42053700
O	3.31233600	0.07518700	-0.34088800
C	4.43138600	-0.80513000	-0.43894700
H	4.45297400	-1.28216100	-1.41783200
H	5.30728300	-0.18166200	-0.29285700
H	4.37267000	-1.57362500	0.33026700
C	-2.75987200	-1.36347200	-0.71117200
H	-2.13475300	-1.81642500	-1.47910100
H	-3.79965900	-1.56340300	-0.96151700
C	-2.45265900	-1.97354000	0.67478100
H	-3.05543900	-1.45376600	1.42251500
H	-2.78836500	-3.01308300	0.65271300
C	-1.00517200	-1.93415600	1.06241700
H	-0.32410700	-2.53740800	0.46693000
C	-0.51604500	-1.21669800	2.06512400
H	-1.16054700	-0.60211700	2.68516300
H	0.53933000	-1.22754100	2.30831600

Mol7-T1_b

C	1.13907500	0.32822500	-0.23475900
C	0.14904600	2.40529500	0.75527200
C	-0.97355100	2.22370300	-0.24842300
C	-0.12933900	-0.02260800	-0.56157600
H	-0.16080100	2.02745200	1.73473700
H	-1.90530100	2.65776900	0.09968700
H	-0.71479700	2.69555700	-1.20094400
H	-0.29167700	-1.03196100	-0.90795800
H	0.39680600	3.45885700	0.86746300

C	1.41053100	1.65605300	0.35457200
C	2.14316600	-0.74819500	-0.43132400
C	-2.52457800	0.38112700	-0.76145200
N	-1.20993800	0.79813800	-0.50233900
O	2.50338800	2.12133300	0.55562200
O	1.87083300	-1.83392500	-0.88778200
O	-3.41945900	1.18916200	-0.73819100
O	3.36576200	-0.41077900	-0.03986700
C	4.35801900	-1.42101000	-0.20065500
H	4.44301500	-1.70513100	-1.24852700
H	5.28396100	-0.98026100	0.15486500
H	4.10036800	-2.30285400	0.38431100
C	-2.80043300	-1.08409300	-1.00894800
H	-2.03671200	-1.56648600	-1.61636600
H	-3.73551700	-1.10235300	-1.56569400
C	-2.99677500	-1.85512000	0.32113300
H	-3.76938600	-1.33760300	0.89467000
H	-3.38607800	-2.84185000	0.05947900
C	-1.75931700	-2.00416800	1.14881600
H	-1.07145300	-2.80692900	0.89691000
C	-1.45903300	-1.13111900	2.26887400
H	-1.76009200	-1.39005300	3.27784900
H	-0.82782400	-0.25598100	2.15845000

S8. References

[ref1] (a) O'Boyle, N. M.; Banck, M.; James, C. A.; Morley, C.; Vandermeersch, T.; Hutchison, G. R. J. Cheminform 2011, 3, 33. (b) Open Babel, version 3.1.1, compiled from source code obtained at https://github.com/openbabel/openbabel
[ref2] Pérez-Soto, R.; Besora, M.; Maseras, F.; pyssian v1.1.0, 2025. maserasgroup-repo/pyssian: 1.1.0 (v1.1.0). Zenodo. https://doi.org/10.5281/zenodo.14801551
[ref3] Bannwarth, C.; Caldeweyher, E.; Ehlert, S.; Hansen, P.; Pracht, P.; Seibert, J.; Spicher, S.; Grimme, S. WIREs Comput. Mol. Sci. 2020, 11, e01493
[ref4] Probst, D.; Reymond, J.-L.; J. Cheminformatics 2018, 10, 66.
